# Computational insights into rational design and virtual screening of pyrazolopyrimidine derivatives targeting Janus kinase 3 (JAK3)

**DOI:** 10.3389/fchem.2024.1425220

**Published:** 2024-08-12

**Authors:** Abdelmoujoud Faris, Ivana Cacciatore, Radwan Alnajjar, Adnane Aouidate, Mohammed H. AL Mughram, Menana Elhallaoui

**Affiliations:** ^1^ LIMAS, Department of Chemical Sciences, Faculty of Sciences Dhar El Mahraz, Sidi Mohamed Ben Abdellah University, Fez, Morocco; ^2^ Department of Pharmacy, University “G. d’Annunzio” of Chieti-Pescara, Chieti, Italy; ^3^ CADD Unit, PharmD, Faculty of Pharmacy, Libyan International Medical University, Benghazi, Libya; ^4^ School of Applied Sciences of Ait Melloul, Ibn Zohr University, Agadir, Morocco; ^5^ Department of Pharmaceutical Chemistry, College of Pharmacy, King Khalid University, Abha, Saudi Arabia

**Keywords:** tyrosine kinase, autoimmune diseases, rheumatoid arthritis, cancer, JAK3

## Abstract

The Janus kinase 3 (JAK3) family, particularly JAK3, is pivotal in initiating autoimmune diseases such as rheumatoid arthritis. Recent advancements have focused on developing antirheumatic drugs targeting JAK3, leading to the discovery of novel pyrazolopyrimidine-based compounds as potential inhibitors. This research employed covalent docking, ADMET (Absorption, Distribution, Metabolism, Excretion, Toxicity) analysis, molecular dynamics modeling, and MM/GBSA (Molecular Mechanics Generalized Born Surface Area) binding free energy techniques to screen 41 in silico-designed pyrazolopyrimidine derivatives. Initially, 3D structures of the JAK3 enzyme were generated using SWISS-MODEL, followed by virtual screening and covalent docking via AutoDock4 (AD4). The selection process involved the AMES test, binding affinity assessment, and ADMET analysis, narrowing down the candidates to 27 compounds that passed the toxicity test. Further covalent docking identified compounds 21 and 41 as the most promising due to their high affinity and favourable ADMET profiles. Subsequent development led to the creation of nine potent molecules, with derivatives 43 and 46 showing exceptional affinity upon evaluation through molecular dynamics simulation and MM/GBSA calculations over 300 nanoseconds, comparable to tofacitinib, an approved RA drug. However, compounds L21 and L46 demonstrated stable performance, suggesting their effectiveness in treating rheumatoid arthritis and other autoimmune conditions associated with JAK3 inhibition.

## Introduction

Rheumatoid arthritis (RA) is a chronic autoimmune disease that affects a large number of people globally (Hitchon et al., 2023; Klebanoff et al., 2023). With millions of people affected each year, the global incidence of RA emphasizes its tremendous impact on public health. According to recent research, this disabling ailment affects around 1% of the global population (Isaifan, 2020; Alharthi et al., 2022). These statistics highlight the critical need to address the illness burden and provide better solutions for people afflicted. RA requires additional examination for a variety of compelling reasons (ScienceDirect, 2022). Literature data report that JAK (Janus kinase) and other related proteins are involved in the pathophysiology and signalling pathways related to RA. They are essential in controlling immunological responses and inflammation, which are key elements in the onset and development of illness. Even though other proteins are also involved in RA, concentrating on JAK and the JAK-related proteins offers the chance to discover novel molecules that could interfere with the biochemical pathways involved in the onset of RA.

Signalling by JAK3 is involved in many cytokines that promote inflammation. Their suppression thwarts their pro-inflammatory activity. These two kinases contribute to T and B lymphocyte activation and proliferation (Henderson Berg et al., 2022). Their inhibition decreases immune system activation in autoimmune disorders. Inhibiting JAK3 reduces the production of pro-inflammatory cytokines such as interleukin-6 and interleukin-17, which are two important cytokines in rheumatoid arthritis (Henderson Berg et al., 2022; Kotyla et al., 2022). JAK3 inhibitors have shown great success in treating rheumatoid arthritis, decreasing inflammation and symptoms (Boyadzhieva et al., 2022; Chen et al., 2023; Sardana et al., 2023). One immunomodulatory method of action gives them a viable treatment choice for instances that are resistant to traditional treatments such as DMARDs and anti-TNF medicines. Many cytokines, including IL-2, IL-4, IL-7, IL-9, IL-15, and IL-21, contain the gamma subunit (Prasad et al., 2023). The binding of these cytokines to their receptors activates the JAK/STAT signalling pathway by phosphorylation of gamma by receptor-associated JAK kinases (Choy, 2019). The interleukins IL-7 and IL-15 activate JAK3, which is responsible for the phosphorylation of gamma 13. This phosphorylation, in turn, creates binding sites for STAT proteins. Once activated, these proteins boost the transcription of genes involved in lymphocyte proliferation and differentiation (Menet, 2018; Morris et al., 2018). Medication that inhibits JAK3 prevents the phosphorylation of gamma caused by related cytokines. This limits the activation of downstream STAT pathways and, hence, the pro-inflammatory immune response, which is driven mostly by T and B cells. JAK1 and JAK3, through gamma phosphorylation, play a major role in the signal transduction of several pro-inflammatory cytokines, explaining their involvement in autoimmune disorders (Menet, 2018). Regulation of these two kinases modulates immune system activation in autoimmune disorders by exerting similar effects on the JAK/STAT pathway. Preventing these proteins can reduce inflammation and delay disease progression, giving an alternative for individuals who do not respond to traditional treatments. The JAKs have an integrated pseudokinase domain (JH2) that modulates the neighboring kinase domain (JH1). The therapeutic targeting of JH2 domains has been less widely investigated and may represent an avenue to control the JAKs without the deleterious effects associated with targeting the nearby JH1 domain (Henry and Jorgensen, 2023; Rodriguez Moncivais et al., 2023). The FDA recently approved this drug, a TYK2 JH2 ligand, to treat plaque psoriasis, demonstrating the potential of this technique. In this light, the structure and targetability of the JAK pseudokinases are discussed, as well as the status of the development of ligands that bind to these domains (Grant et al., 2023; Henry and Jorgensen, 2023). The known approach to developing small-molecule JAK3 inhibitors is to target the JAK3 kinase domain’s. There’s four JAK protein, JAK1, JAK2, JAK3, and TYK2. JAK consists of a FERM domain, an SH2-linked domain, a kinase domain, and a pseudokinase domain. The kinase domain is essential for JAK function because it allows JAKs to phosphorylate proteins (Christy and Shankari, 2020). Information conveyed by the JAK3 protein influences the formation and maturation of white blood cells known as T lymphocytes, B lymphocytes, and natural killer cells, which are important for immune system regulation. Tofacitinib is a drug used to treat specific autoimmune illnesses, including rheumatoid arthritis (Yang et al., 2021). It works by regulating the immune system to minimize inflammation related to certain illnesses (Hosseini et al., 2020). Tofacitinib has been approved by numerous regulatory bodies, including the Food and Drug Administration (FDA) in the United States, for the treatment of rheumatoid arthritis and other autoimmune illnesses (Mogul et al., 2019; Roskoski JrR., 2023a).

**FIGURE 1:**
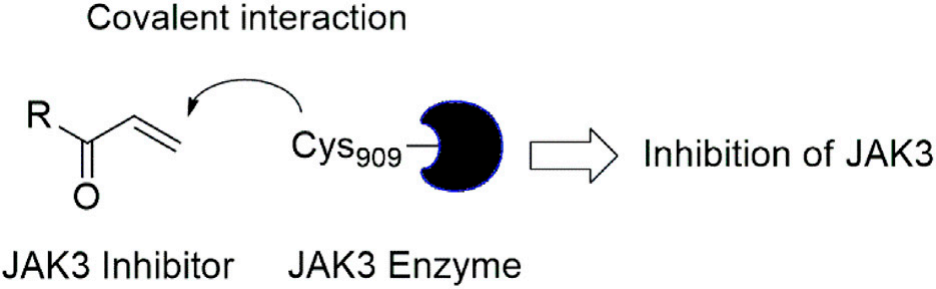
Mechanism of irreversible inhibition by addition of Cys_909_ residue to acrylaldehyde.

In this study, JAK inhibitors, especially JAK3 inhibitors, are designed particularly to target JAK3, a protein that is crucial to the development of RA. The danger of interfering with unrelated cellular processes is reduced by carefully controlling JAK3 signalling, potentially lowering side effects and improving treatment specificity. The Cys_909_ residue of JAK3 is significant because it plays a critical role in the binding affinity and specificity of JAK3 inhibitors. JAK3 inhibitors must bind with Cys_909_ residue to inhibit JAK3 activity and subsequent signalling processes that contribute to the pathophysiology of RA. Previous studies demonstrated that selective and covalent JAK3 inhibitors target the Cys909 residue through a covalent link via a 1,4-Michael addition process with an acryl group (Figure 1) (Tan et al., 2015; Castelo-Soccio et al., 2023). This covalent alteration improves the inhibitors’ potency and selectivity, resulting in a more effective and targeted suppression of JAK3 activity. Taking into account these data, the acrylaldehyde group was considered a key structural element in the *in silico* design of our inhibitors for different reasons: a) the link formed between the inhibitor and the Cys_**909**_ residue of JAK3 enzyme is covalent and hardly dissociable; b) the covalent interaction interrupt JAK3’s functions and downstream signalling pathways permanently; c) the introduction of the acrylaldehyde group allows the design of inhibitors with tailored properties, enhanced efficiency, and lowered off-target effects. We focused on the *in silico* design of novel JAK3 inhibitors based on the pyrazolopyrimidine scaffold bearing the acrylaldehyde function (Shankar et al., 2022; Caveney et al., 2023). Pyrazolopyrimidine derivatives are aromatic nitrogen heterocycles with a wide range of biological activities such as antitumor, antimalarial, antiparasitic, insecticidal, antirheumatic, antibacterial, antifungal, cardioprotective, antiviral, anti-inflammatory, and antioxidant (Upadhyay, 2017; Namdeo et al., 2023). Drugs containing the pyrazolopyrimidine scaffold are currently marketed and prescribed to people who have insomnia. However, some new derivatives produce anxiolytic effects with relatively little sedation and are being developed for use as anti-anxiety drugs without sedative effects (these drugs include Zaleplon–hypnotic (Sonata^®^), Indiplon–hypnotic, Ocinaplon–anxiolytic, and Lorediplon–hypnotic).

Using the Structure-Based Virtual Screening (SBVS) approach, this research aimed to discover JAK3 inhibitors endowed with more potency and selectivity against JAK3 enzyme. The newly developed compounds demonstrated high molecular activity by engaging with their target. These compounds include sulfonamide groups, which are still being researched and used today, despite their difficulties in synthesis. However, with the means available today, we can achieve their synthesis due to their potent effects against autoimmune diseases (Ouyang et al., 2016; Mushtaq and Ahmed, 2023; Lim et al., 2024). In this pilot study, the pkCSM and ADMELab 2.0 tools (Azzam, 2023) were used to perform the ADMET analysis of 41 designed molecules. Compounds with no toxicity (resulted negative to the tests of AMES toxicity) were subjected to a second screening using AutoDock 4.2 software (AutoDock4 and AutoDockTools4, 2022). In addition, molecular dynamics simulations were performed using the GROMACS package (Abraham et al., 2023) for 300 ns to verify the stability of the best complexes. Finally, the binding free energy of two complexes was evaluated using the MM/GBSA method (Valdés-Tresanco et al., 2021). In the realm of Computational-Aided Drug Design (CADD), the objective is to strategically design 41 guides to pinpoint the top two molecules, each with its corresponding affinity. Through meticulous analysis, the development of these two molecules unfolds, revealing novel compounds boasting heightened potency and remarkable affinity. This enhancement is attributed to the essential incorporation of SO2. Consequently, the newly designed molecules exhibit notable stability during molecular dynamics simulations with MM/GBSA, suggesting their potential candidacy for *in vivo* and *in vitro* studies. Building upon the success of CADD as a guiding principle for prior *in vivo* and *in vitro* investigations, these newly identified molecules emerge as promising subjects for further exploration in the field (Al-Karmalawy et al., 2023).

## Methods and materials

### Homology modeling (model building)

When experimental structures are lacking, homology modeling emerges as a key tool in the field of protein structure determination (Abrigach et al., 2018). This technique consists of multiple consecutive processes, including template selection, target template alignment, model creation, validation, and assessment. In our study, the model creation phase used a variety of tools, with a focus on the SWISS-MODEL server (Nikolaev et al., 2018), to improve accuracy and dependability.

**FIGURE 2:**
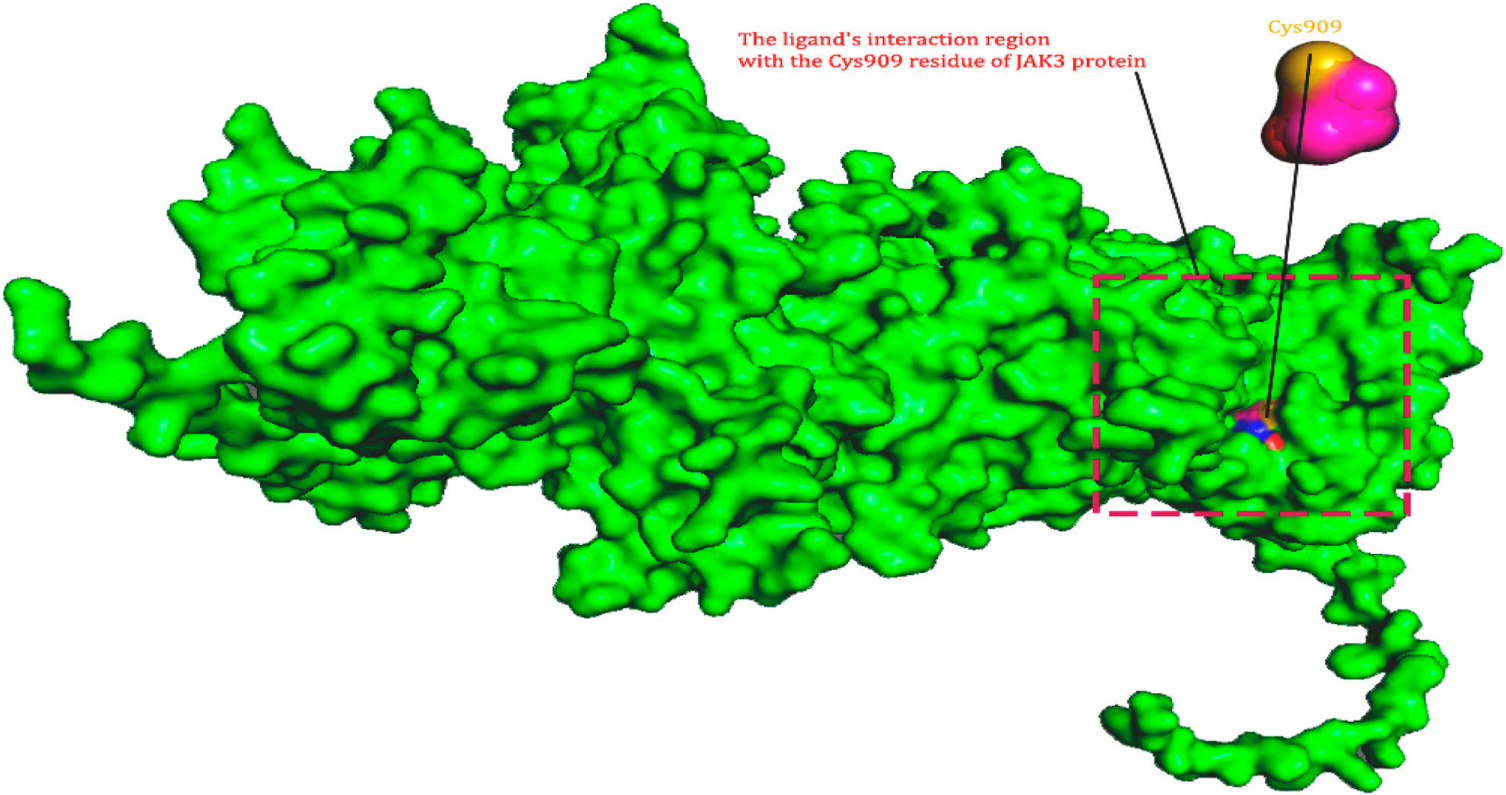
The ligand’s interaction region with the Cys909 residue of JAK3 protein.

In this investigation, we used the Uniprot public domain protein database (http://www.uniprot.org/) to identify the protein sequence of Tyrosine-protein kinase JAK3 (P52333 JAK3 HUMAN, FASTA format (Figure 2) (JAK, 2022). P52333 was chosen as the target since it is one of the enzymes involved in the breakdown of host hemoglobin. As a result, significant crystallographic investigations on P52333 with different inhibitors have been done, opening the path for the application of computational approaches to investigate potential anti-rheumatoid arthritis medicines against JAK3 (Simoncic et al., 2002). Using the SWISSMODEL service, the acquired sequence was used to predict a 3D homology model of the Tyrosine-protein kinase JAK3 protein (betacoronavirus, 2019). The Basic Local Alignment Search Tool (BLAST) was used to find homologs that might be used as templates (Homology Modeling and Docking Studies, 2022). The templates were chosen because of their excellent quality, lack of mutations, strong match of ligand structure to experimental data, and resemblance to the target. As a result, the best template receptor (PDB: 4Z16) was chosen as the reference template (Tan et al., 2015). Once the model’s 3D structure was created, it was critical to analyse and certify its correctness.

### Evaluation and validation of the retained homology model

A crucial stage in forecasting protein structures is evaluating the accuracy of protein structure models. In this work, the Ramachandran plot (Ho and Brasseur, 2005), a useful tool for evaluating protein structures and showing the dihedral angles (Ψ and φ) of amino acid residues, was generated using the SAVES web server. Based on these angles, the plot offers insights into various polypeptide conformations (Homology, 2022). Additionally, to raise the model’s quality significantly, we used the Qualitative Model Energy Analysis (QMEAN) score tool. This parameter enables us to separate superior homology models from worse ones (Benkert et al., 2008). We used the BIOVIASTUDIO program (Systèmes, 2020) to see the protein’s structure. Considering previous research on JAK3 inhibition as a treatment for rheumatoid arthritis, the active site in the protein generated by homology modelling for the covalent docking between ligand and protein, specifically through a covalent link with Cys909, bears important significance. These results emphasize Cys909s crucial function in regulating the activity and specificity of the protein-ligand interaction, particularly when targeting the JAK3 pathway for RA treatment. The discovery and characterization of this active region offer important insights for the design and development of new drugs that target JAK3 more effectively and selectively to cure this crippling condition.

### Molecular docking

Standard molecular docking and covalent molecular docking are two different techniques used to study the interactions between a ligand and a protein. Standard molecular docking relies on the use of algorithms to predict the most favourable conformation of the ligand-protein complex based on binding energy. Covalent molecular docking, on the other hand, considers covalent interactions between the ligand and the protein. In this case, the ligand is designed to contain a reactive function (warheads) that can form a covalent bond with a specific residue of the protein. This method is beneficial for studying enzymes and proteins involved in diseases, as it allows for specific targeting of the active residues of the protein.

### Molecular docking of tofacitinib drug

Prior to molecular docking, the ligands were optimized to be docked using Avogadro software. Next, the JAK3 structure was downloaded from the RCSB database (PDB ID: 4Z16). The 4Z16 crystal complex contains the co-crystallized ligand 4LH, the protein 4Z16, and the co-crystal water molecules. The protein was prepared by removing all water molecules followed by protonation (adding hydrogens at pH = 7.00) to the JAK3 protein structure using Discovery Studio software. The active site of 4Z16 is defined by the sphere containing the co-crystallized ligand (4LH). Once the ligand and protein were prepared, we performed molecular docking using AD4 and AutoVina. The three-dimensional grid was defined using the AUTOGRID algorithm, which determines the binding energy of ligands with their receptor (Morris et al., 2008). The default grid size is x = 60, y = 60, and z = 60, with a distance between grid points of 0.375 Å (Morris et al., 2008). The centre of the grid is the active site of the receptor with coordinates (x = −6.68875 Å, y = −14.7757 Å, and z = 1.89597 Å). The docking results obtained by AD4 and Vina were visualized using Discovery Studio software. CovDock (Covalent Docking), the guide for the procedure, explains several actions that may be taken to complete the covalent docking approach utilizing AD4 with flexible side chains (Bianco et al., 2016).

### Covalent docking of new design compounds

The ligand alignment must first be constructed using the available Covalent.py script, and the ligand structure must be modelled with a piece of the alkylated residue already present. By defining the atom indices in the ligand file or by specifying a SMARTS pattern and the alignment atom indices in the pattern itself, the ligand alignment may be specified. The ligand that has been covalently attached to the residue is the process output. It is significant to remember that before beginning the covalent docking procedure, the ligand structure may need to be decreased in addition to the preceding procedures. Software like Avogadro (Rayan and Rayan, 2017) and the MM2 force field can be used for this. The prepareCovalent.py script may then be run with the reduced structure as an input to create the ligand alignment. Second, PDBQT files for the receptor structure and the covalent ligand must be created. The prepare receptor4.py script may create the default PDBQT files for AutoDock, provided MGLROOT is set up and installed. Thirdly, the stiff and flexible components that will be utilized for docking are generated using PDBQT files. Using the prepare_flexreceptor4.py script, the stiff portion of the receptor is extracted by indicating which residue should be made flexible. The processed ligand is treated in the same manner. Fourth, parameter files are created for the actual computation. The prepare gpf4.py script is used to build the GPF for AutoGrid, while the prepare dpf4.py script is used to generate the DPF for AutoDock. To ensure that the docking score accurately reflects the interaction between the flexible residue (the ligand) and the stiff receptor, the proper energy model must be explicitly defined in the DPF file. The output produced at each stage of the process is available in the output directory, and AutoGrid and AutoDock may be performed using the regular technique. The preparation of the ligand alignment, creation of PDBQT files, creation of stiff and flexible components, preparation of parameter files, and execution of AutoGrid and AutoDock are all included in the covalent docking approach. You will find the exact parameterization necessary for the ligand covalently attached to the protein in our article. Since the Cys909 residue is covalently attached to the ligand, a distinct force field is needed for the computations (Faris et al., 2024a).

In this study, our primary focus was on predicting new compounds containing an acrylaldehyde moiety that would favour an irreversible covalent bond with the Cys909 residue. For this reason, we employed covalent docking and compared the results with an FDA-approved medication, Tofacitinib, which exhibits potent inhibition of JAK3 but does not form an irreversible covalent bond that dissolves upon binding to JAK3. We chose Tofacitinib for comparison because it provides a suitable reference point due to its known biological activity and lack of irreversible covalent bonding.

### ADMET study

To define the physicochemical characteristics and ADME properties of compounds, their SMILE strings were uploaded to ADMETlab 2.0 and pkCSM web programs (Xiong et al., 2021; Azzam, 2023), respectively. The pkCSM web program (Absorption, Distribution, Metabolism, Excretion, and Toxicity) is a computer program that predicts the pharmacokinetic features of small-molecule medicines. The procedure may be found on the webpage online (pkCSM, 2024). ADMET predicts critical factors such as oral bioavailability, plasma protein binding, and tissue distribution using a mix of mathematical models and databases, allowing researchers to swiftly assess a molecule’s potential as a therapeutic candidate (Faris et al., 2023a; Faris et al., 2023b). This strategy can greatly speed up drug development by reducing the need for animal and clinical trials, as well as save money by detecting problematic compounds early in the development process (Shit and Banka, 2019; Lane et al., 2020; Parvathaneni et al., 2023; Shi et al., 2023).

### Molecular dynamics simulation

GROMACS is a popular and capable molecular dynamics simulation software suite (Abraham et al., 2023). The CHARMM GUI may be used to conveniently customize the preparation of input files for GROMACS simulations (Graphical User Interface) (CHARMM, 2024). The temperature and solvent conditions in the simulation may be specified using this tool. In our investigation, we simulated a complex in solution at a constant temperature of 310 K. The solvent was neutralized by sodium chloride ions and solvated in a 10 cubic box around the protein with the TIP3P water model. To build the protein-ligand combination for MD simulation, the CHARMM36 force field was used in conjunction with the CHARMM GUI (Brooks et al., 2009; Faris et al., 2024b; Faris et al., 2024c). A typical simulation begins with a minimization stage in which the system is relaxed to a local energy minimum. 200,000 minimization steps are taken in this situation. An equilibration phase in which the system is brought to temperature and mechanical equilibrium is established. This equilibration is done in two stages, with a 2 ns initial equilibration and a 5 ns final equilibration, both at a time step of 0.001 ps After equilibration, the simulation’s manufacturing phase may begin. This phase lasts 300 ns and is used to propagate the system’s molecular dynamics as well as sample and evaluate the characteristics of interest. The findings of a GROMACS simulation give significant insights into the behaviour of biological molecules and their interactions, making it a useful tool in a variety of fields of study.

Dynamics metrics in Supplementary Table S1 include RMSD, measuring the deviation of MD simulated structures from the initial configuration, indicating stability. RMSF highlights residue flexibility, RoG assesses protein compactness, and SASA monitors solvent accessibility. DSSP analyses secondary structure composition, while PCA extracts key components governing molecular dynamics. The average distance between atom locations in the simulated structure and the initial reference structure is measured by RMSD. It is an indicator of how far the molecular dynamics (MD) simulated structure has deviated from its initial configuration. A system with a lower RMSD value has less structural drift and is, therefore, more stable. During the MD simulation, RMSF estimates each residue’s variations around its average location. Higher RMSF values indicate that the residues are more flexible. Finding flexible and stiff protein regions may be done with the help of RMSF. The protein’s mass dispersion around its centre of mass is measured by RoG. It may identify the protein structure’s expansion and compactness. The structure may be folded or compacted if RoG is reduced, whereas compaction or unfolding may be indicated by an increase in RoG. The protein’s surface area that is accessible to the solvent is referred to as its “solvent-accessible surface area (SASA). Changes in SASA may be a sign of protein-ligand interactions, folding, ligand binding, or conformational changes. SASA is frequently used to examine the dynamics and stability of proteins. Each residue is given a secondary structure by the DSSP algorithm by its phi/psi angles and hydrogen bonding patterns. It is employed to examine the secondary structure’s composition and evolution during an MD simulation. For instance, alterations in the amount of beta- or alpha-sheets might shed light on the dynamics, folding, and stability of proteins. The free energy landscape governs the thermodynamics and kinetics of molecular activities within a solution. Principal Component Analysis (PCA) is a linear transformation method that extracts key components from data using a covariance matrix or a correlation matrix (normalized PCA). These matrices are built using atomic coordinates to describe the protein’s available degrees of freedom (DOF), such as Cartesian coordinates that define atomic displacements in each conformation.

### Free binding energy (MM/GBSA)

The binding free energy (Eq. 1) may be used to calculate the affinity of receptors for small ligands. The gmx_MMPBSA tool was used to determine the binding free energy (Valdés-Tresanco et al., 2021), this corresponds to the most recent version of gmx_PBSA recognized for its performance for all guides, which you may examine (https://valdes-tresanco-ms.github.io/gmx_MMPBSA/v1.5.6). Using the molecular mechanics/generalized Born surface area (MM/GBSA) approach (Kumari et al., 2014; Valdés-Tresanco et al., 2021). The calculation Equations 1–7 that were employed in this investigation are listed below (Valdés-Tresanco et al., 2021): The total energy change represents the overall stability or instability of the protein-ligand complex in its environment. Each term is computed using the relevant equation, which is detailed in the following (Supplementary Table S2).

$$\Delta_{\text{TOTAL}}=\Delta_{\text{VDWAALS}}+{\Delta E}_{\text{EL}}+{\Delta E}_{\text{GB}}+\Delta_{\text{ESURF}}+{\Delta G}_{\text{SOLV}}$$ (1)

## Results

### Dataset and *in silico* design of novel compounds

**FIGURE 3:**
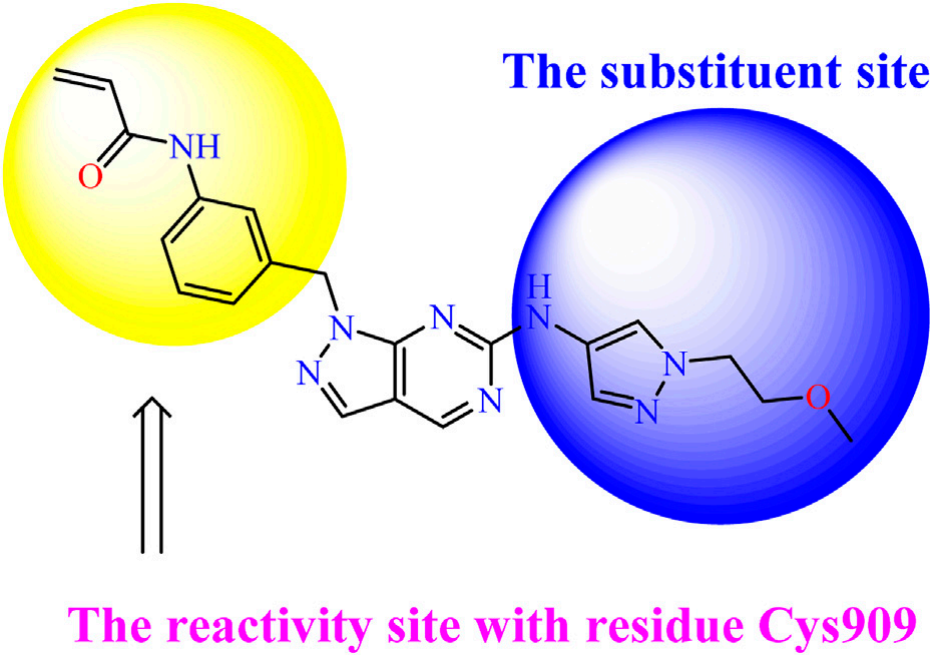
Chemical structure of compound Pyrazolopyrimidn Fusion, a potent JAK3 inhibitor.

Recently, as shown in Figure 3, the compound (N-(3-((6-((1-(2-methoxyethyl)-1H-pyrazol-4-yl)amino)-1H-pyrazolo[3,4-d]pyrimidin-1-yl)methyl)phenyl)acrylamide), in a novel series including new derivatives of pyrazolopyrimidine, is a series of isomeric heterocyclic chemical compounds with the molecular formula C6H5N3. They form the central core of a variety of more complex chemical compounds, including some pharmaceuticals and pesticides. It has been characterized as a potent and selective inhibitor of JAK3, with an IC50 value of 0.1 nM, corresponding to a pIC50 value of 10 (Yin et al., 2020; Faris et al., 2023a; Faris et al., 2023b). Using this scaffolding as a basis, a high-speed technique was used to accelerate the creation of 41 new molecules reported in 2.

### Homology modeling of protein sequence

#### Template selection

Through a covalent interaction with the residue Cys909, which is crucial for inhibiting JAK3, this work focused on JAK3. Target P52333 JAK3 HUMAN was used (Bergmann et al., 2014; Bodaar et al., 2022; Bodaar et al., 2022; Roskoski R., 2023b). It is essential to define the protein’s structure using homology modelling before researching ligand-receptor interactions. The UniProtKB database provided the P52333 JAK3 HUMAN target’s main amino acid sequence. The models were chosen based on their high target similarity, high resolution, and well-documented modelling applicability. Due to the PDB structure 4Z16 greater goodness of fit to experimental data and lack of mutations, it was selected as a viable model.

### Alignment and model building

**FIGURE 4:**
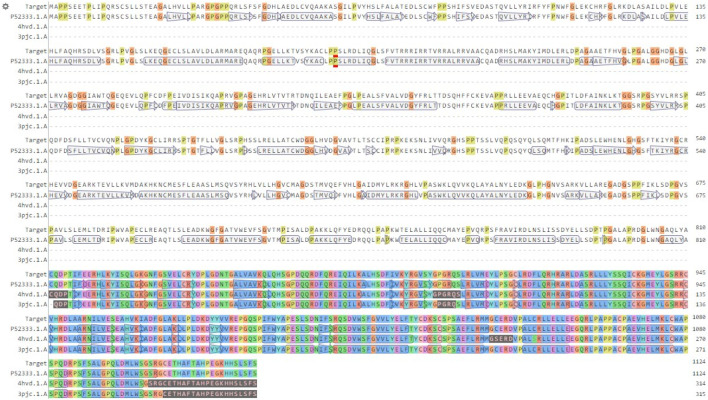
Alignment of the model sequence with the chosen template (PDB: 4Z16).

The target sequence was matched with the model (PDB: 4Z16) using the BLAST alignment tool to find the best alignment with a high level of sequence identity. Consequently, we discovered a model with a similarity of 62%, suggesting its robust and dependable character (Figure 4). Following that, we used the Discovery Studio 4.5 program to display the model’s final three-dimensional structure and locate its active site. This model was also validated using the Ramachandran plot and major chain characteristics acquired from PROCHECK (Abrigach et al., 2018).

### Ramachandran plot and structural quality analysis

**FIGURE 5:**
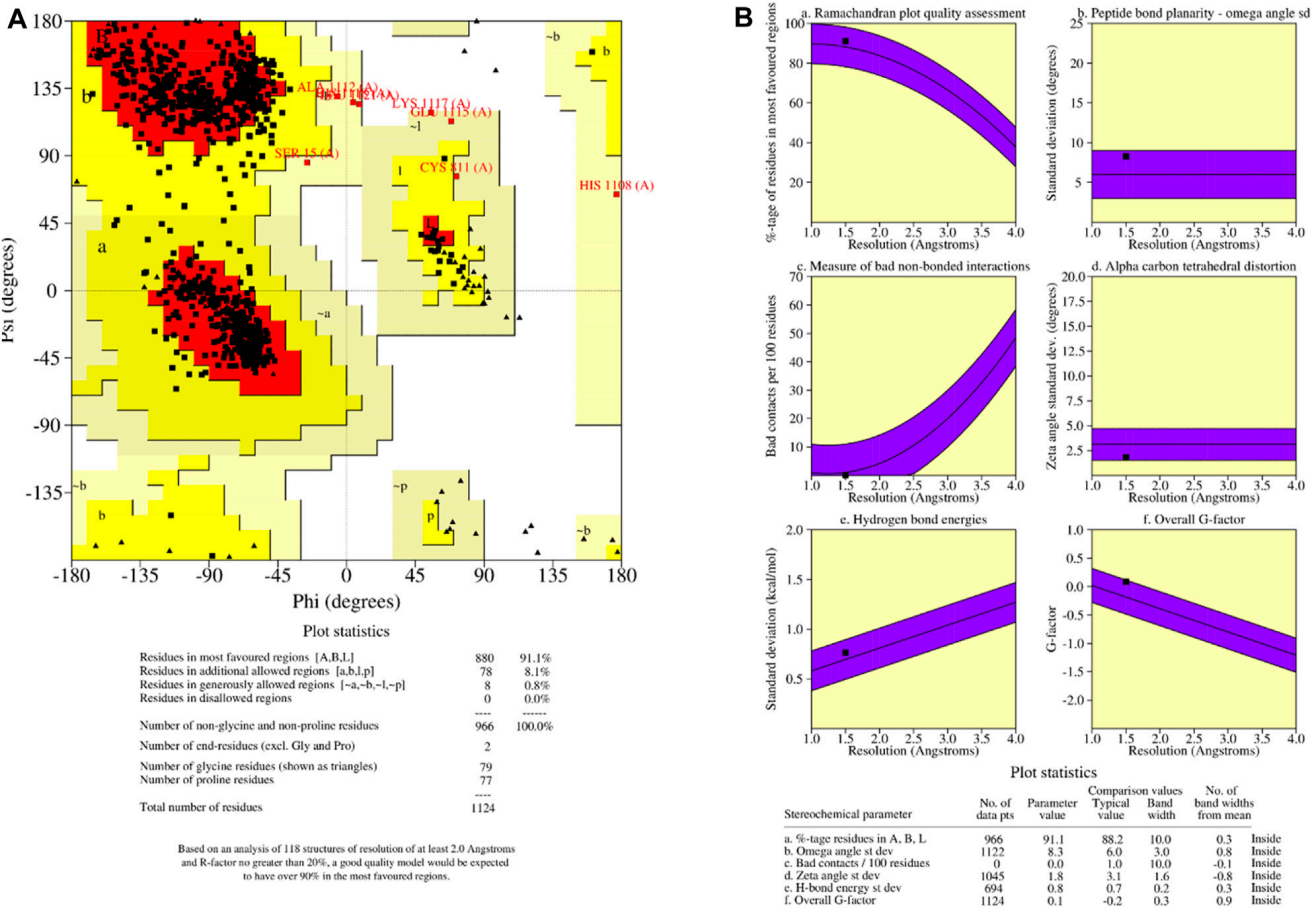
**(A)** Ramachandran plot of predicted protein; **(B)** main-chain parameters obtained by PROCHECK.

It is critical to check the final model before beginning any docking analysis to identify any potential flaws or variations from the usual protein structure. To assess the quality of the predicted protein structure, the ProCHECK tool was used. The calculation of phi-psi torsion angles for each residue produced a Ramachandran plot, which revealed that 91.1 percent of amino acid residues were in the core regions (red color), 8.1 percent were in additional allowed regions (yellow color), and 0.8 percent were in generously allowed regions (light yellow color) (Figure 5A). In contrast, only 0% of residues were discovered in banned areas due to their substantial distance from the enzyme’s active site (white color). The measured parameters of our homology model (solid squares) fell within the dark band in each plot, representing well-refined structure results based on the six properties (Figure 5B): (a) Ramachandran plot quality, (b) peptide bond planarity, (c) unfavourable non-bonded interactions, (d) C tetrahedral distortion, (e) main-chain hydrogen bond energy, and (f) overall G-factor versus resolution. Based on these findings, we can certainly infer that our model corresponds to the parameters of a stable design. The validation results from the Ramachandran plot and main-chain parameter plots encourage us to assume that our model for P52333 JAK3 HUMAN was sufficiently dependable to perform ligand-protein docking study. The Ramachandran plot and main-chain parameter plot validation results led us to conclude that the model we generated for P52333 JAK3 HUMAN was sufficiently dependable to perform ligand-protein docking investigation in our study (En-nahli et al., 2022; Mahjoubin-Tehran et al., 2022).

QMEAN analysis was used to guarantee the data’s correctness. QMEAN assesses the quality of a protein model by combining three structural descriptions. The QMEAN Z-score assesses the similarity between the structural characteristics of the modeled protein and those seen in experimental structures (Saih et al., 2021).

Table 1 shows the findings for the various scoring function measurements, with related Z-scores: QMEAN: The overall score is 0.67 0.05, suggesting that the structure’s quality is typically rated positively. QMEAN Z-score: 0.49, indicating that the analysed structure deviates somewhat from the predicted distribution. C interaction energy Z-score: The score is −0.63, showing that the C interaction is less common than predicted. The Z-score for every atom pairwise energy is 0.27, indicating that this energy is somewhat greater than predicted. The solvation energy Z-score is −0.07, suggesting that the solvation energy is somewhat lower than predicted. Torsion Z-score: 0.37, indicating that the torsion is slightly beyond the normal range when compared to the reference distribution. Finally, these results reveal that the examined structure is generally of good quality, while elements such as C interaction and torsion deviate from the predicted average. These findings must be considered when interpreting the overall quality of the examined structure.

**TABLE 1:** Z-scores of QMEAN for generated homology model.

Scoring function term	Z-score
C_β interaction energy	−0.63
All-atom pairwise energy	0.27
Solvation energy	−0.07
QMEAN score	0.67

### *In silico* evaluation of the toxicity of designed compounds

**FIGURE 6:**
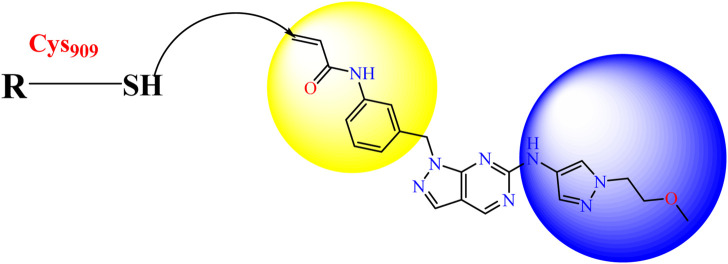
Structural modifications and pharmacological assessment.

We conducted an *in silico* design of 41 molecules, as detailed in Table 2. The initial screening utilized the Ames toxicity index, leading to the identification of 27 non-toxic molecules. The design of novel molecules considered structural elements from known compounds with favourable pIC_50_ values of 10 (Figure 6). These molecules could form covalent bonds, specifically through acrylaldehyde (depicted in yellow), targeting the Cys909 residue. The blue regions were modified by combining various substituents known for their steric, electrostatic, hydrophobic, and hydrogen bond donor/acceptor properties. These modified structures underwent molecular docking simulations to select molecules with a high affinity (expressed in Kcal/mol), devoid of Ames toxicity and possessing suitable pharmacological characteristics. To validate molecular stability, we employed molecular dynamics simulations, a potent approach demonstrated effectively in previous impactful studies for the synthesis of new molecules.

**TABLE 2:** Chemical structures of the *in silico* designed molecules.

No	2D	AMES toxicity
1	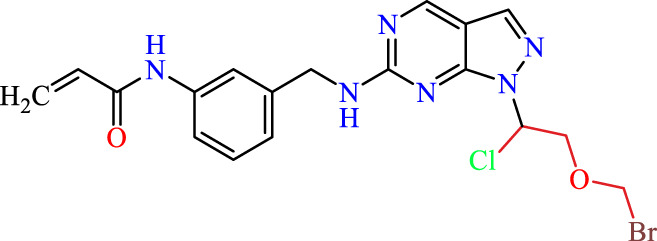	Yes
2	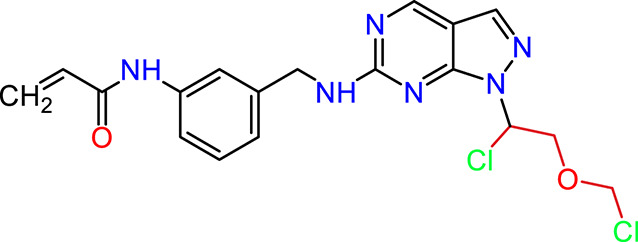	Yes
3	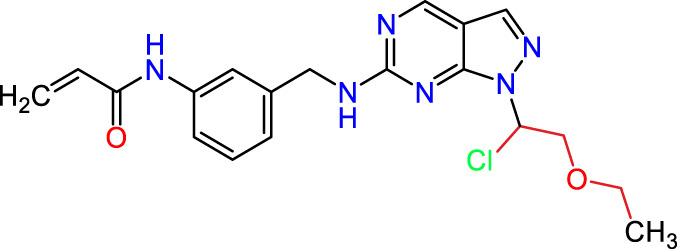	Yes
4	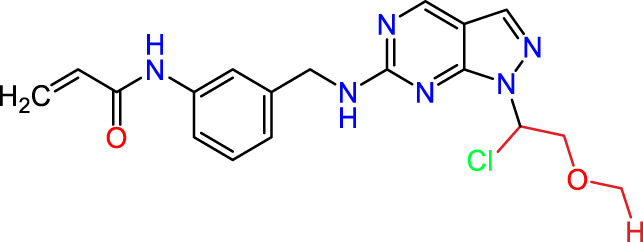	Yes
5	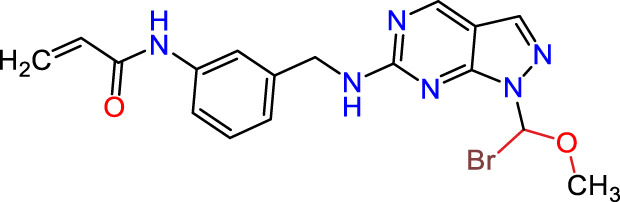	Yes
6	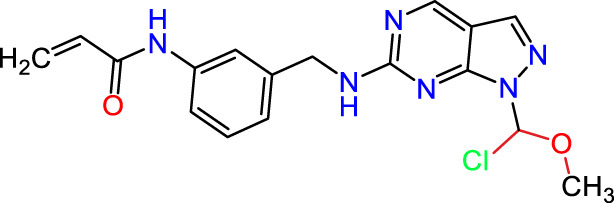	Yes
7	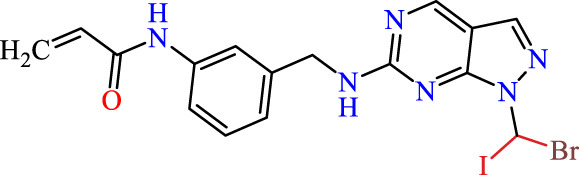	Yes
8	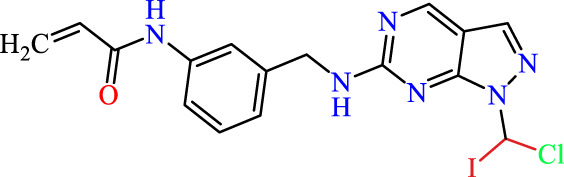	Yes
9	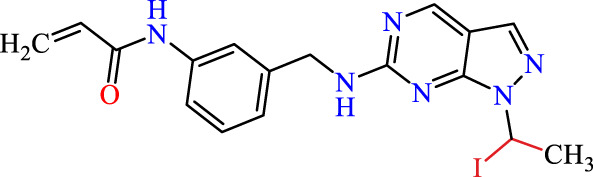	Yes
10	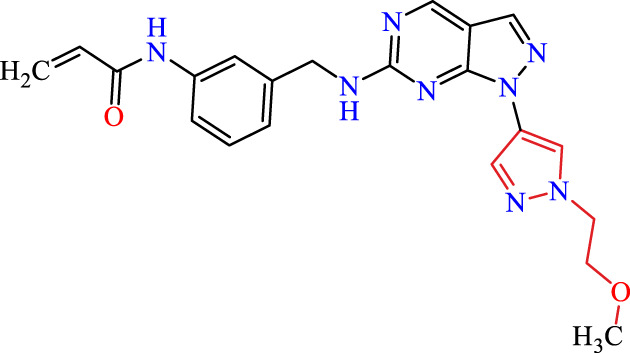	No
11	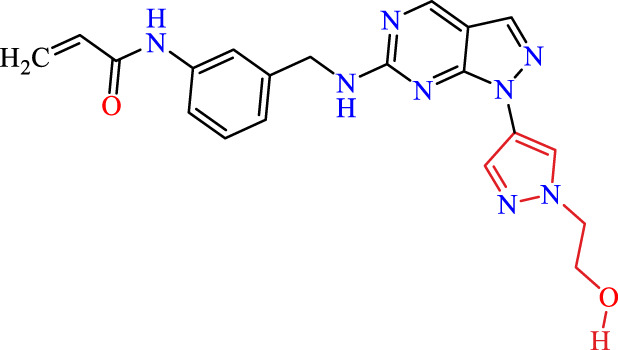	No
12	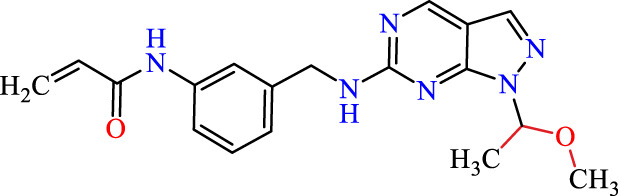	No
13	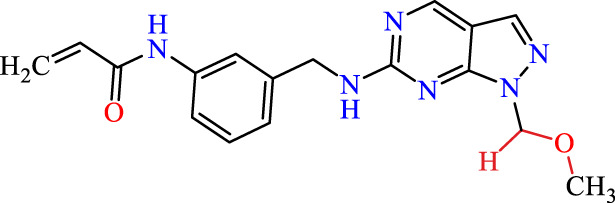	No
14	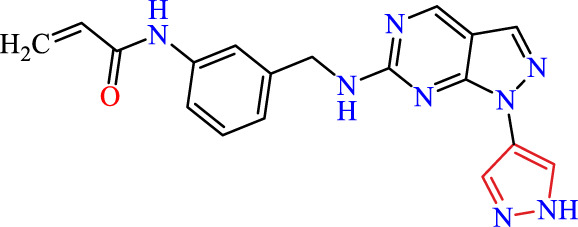	No
15	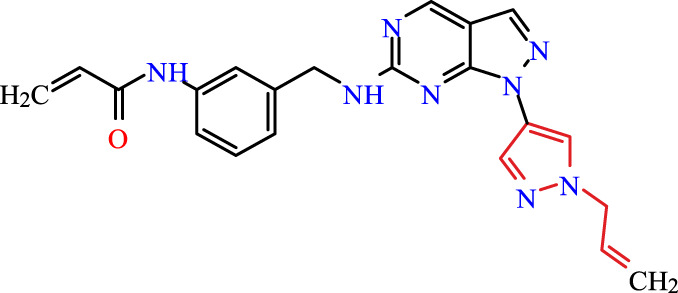	No
16	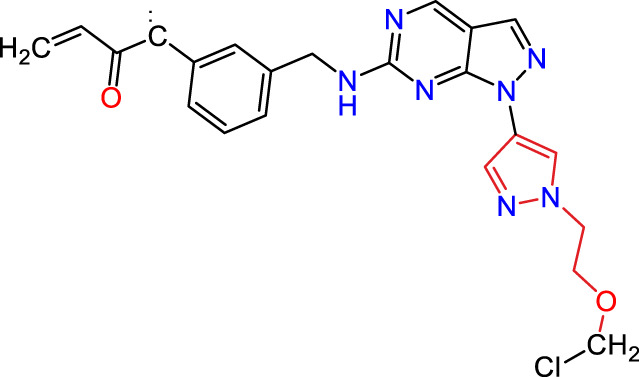	No
17	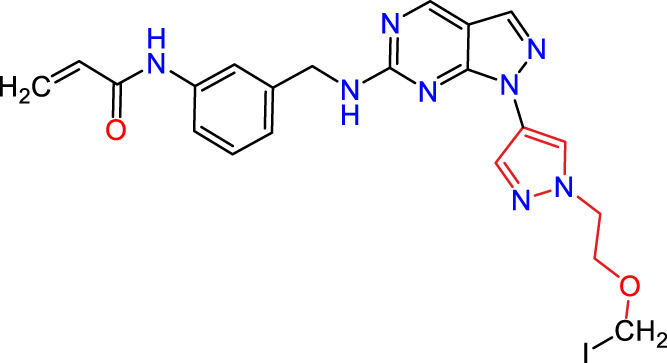	No
18	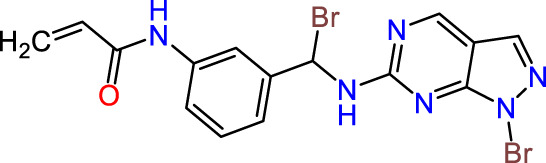	No
19	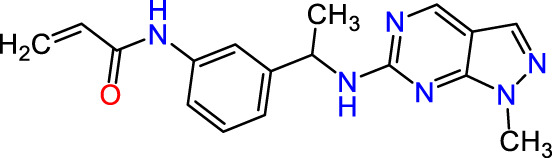	No
20	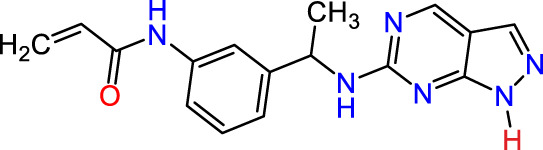	No
21	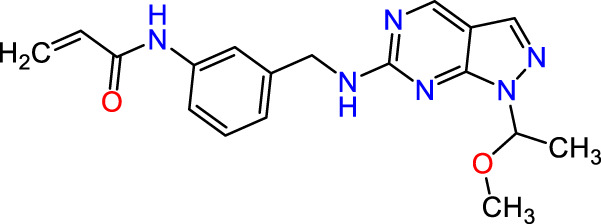	No
22	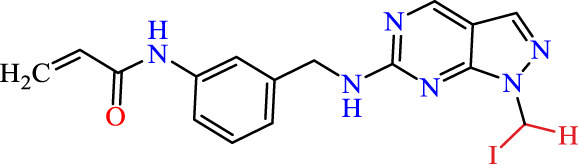	Yes
23	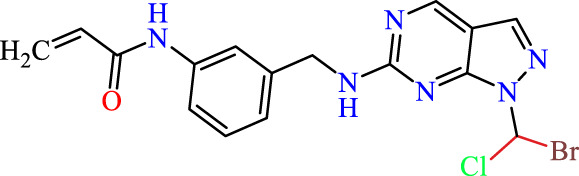	Yes
24	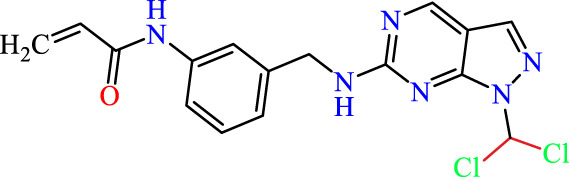	Yes
25	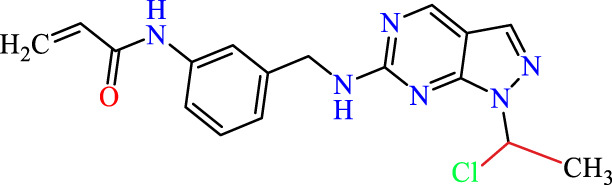	Yes
26	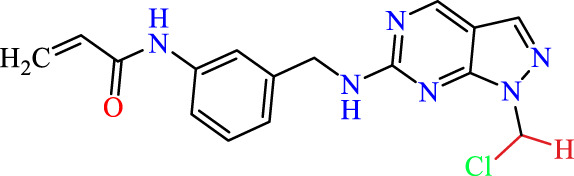	Yes
27	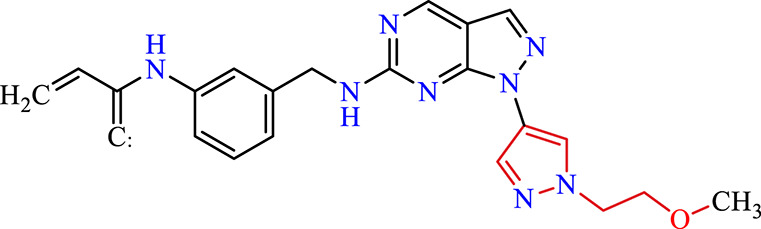	No
28	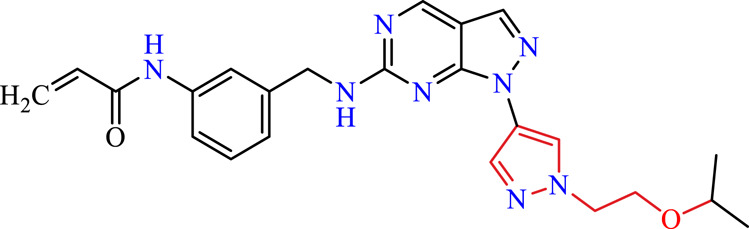	No
29	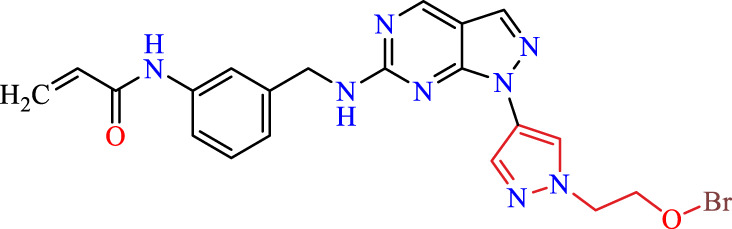	No
30	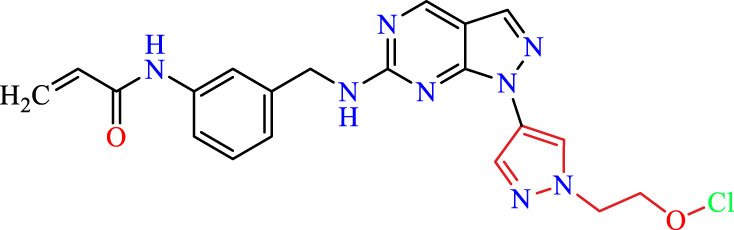	No
31	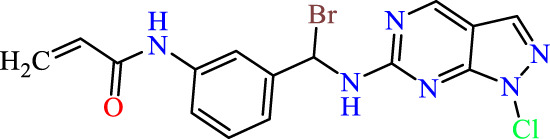	No
32	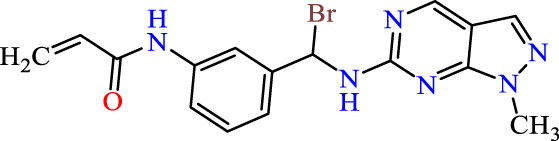	No
33	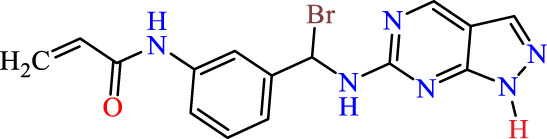	No
34	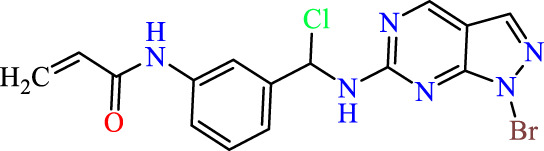	No
35	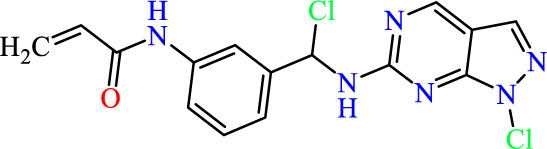	No
36	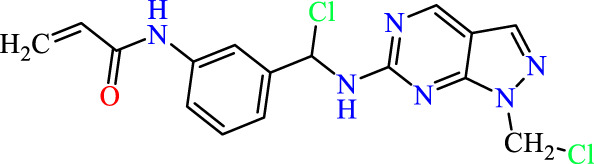	No
37	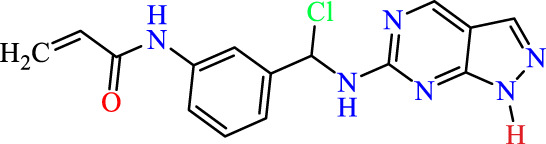	No
38	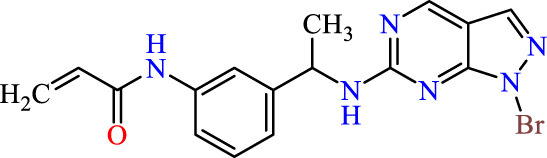	No
39	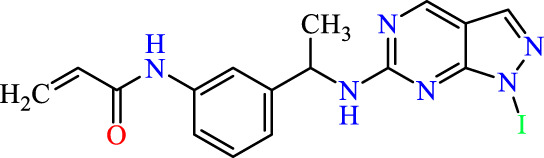	No
40	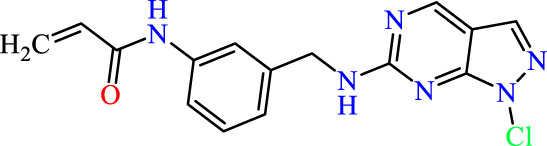	No
41	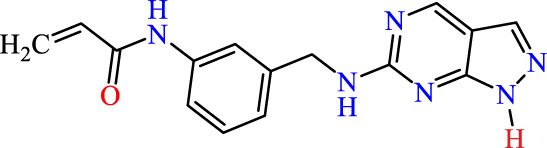	No
42	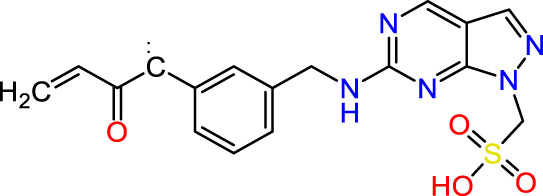	No
43	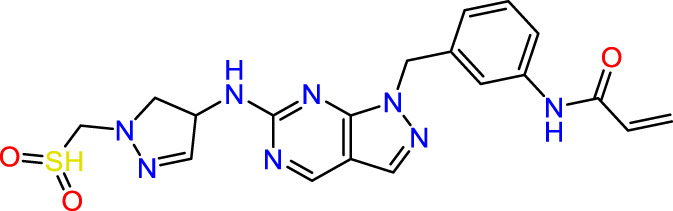	No
44	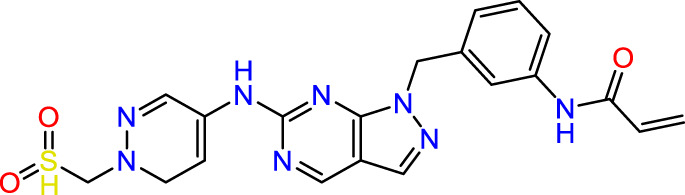	No
45	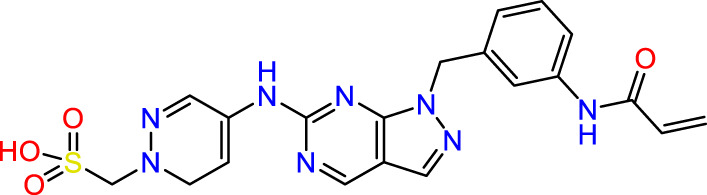	No
46	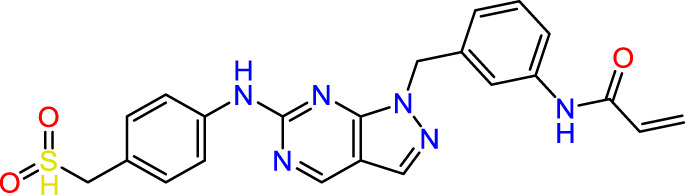	No
47	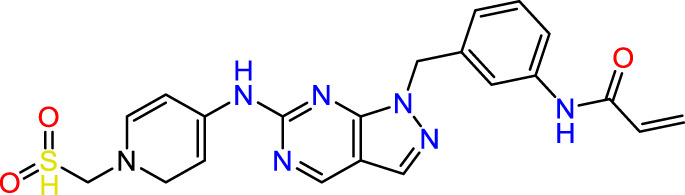	No
48	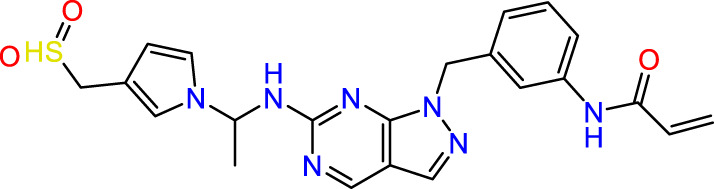	No
49	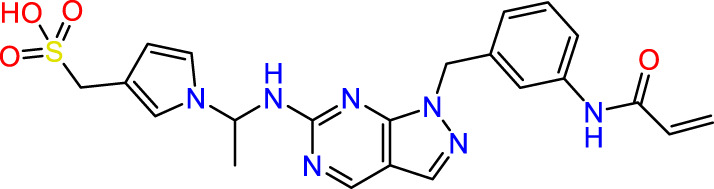	No
50	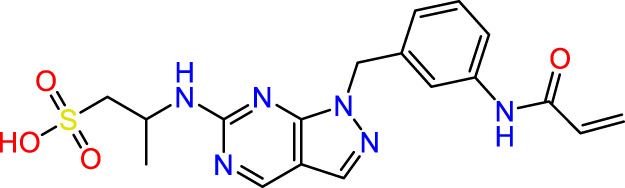	No

Thus, a covalent docking technique was used in the following investigation of the 27 non-toxic derivatives. Covalent docking is a molecular modelling technique that predicts probable covalent binding sites between a small chemical and a target protein. This method is very useful in drug design since it allows for the precise targeting of active amino acid residues in the target protein. Covalent docking allows for a more extensive examination of the selected 19 molecules and their potential for interaction with the target protein. This stage will give us a better idea of how these compounds could interact with the target and forecast their efficacy as prospective medications.

### Redocking analysis

**FIGURE 7:**
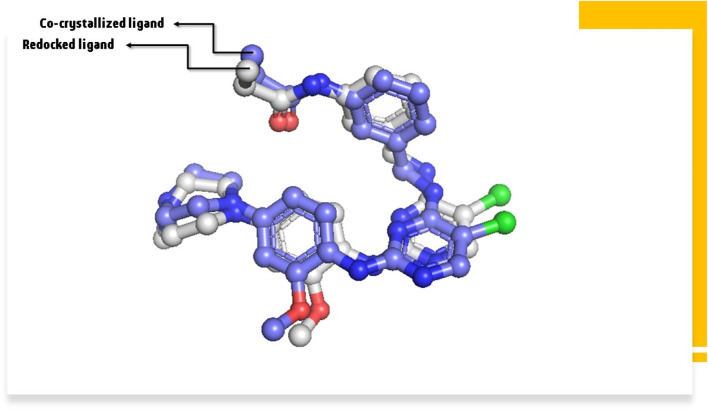
The RMSD between co-crystallized ligand and redocked ligand.

As the setup of molecular docking, redocking is the process of repositioning a ligand that has been temporarily withdrawn from a protein binding site. The RMSD is commonly used to determine how closely the redocked pose matches with the crystallographic conformation or a reference conformation. The RMSD of 0.96 Å before redocking (Figure 7) suggests a close match between the redocked position and the reference conformation. In other words, the structure of the relocated ligand is very similar to that obtained experimentally or from a reference posture. Lower RMSD indicates higher structural agreement. This suggests that the redocking approach has been successful and is widely regarded as a good match.

Covalent Docking Screening.Afterward, the molecules selected on 5, representing AMES toxicity, underwent a covalent docking study. Following covalent docking, molecules **41** and **21,** with the highest affinity, were selected for analysis and future investigation. To evaluate the pharmacokinetic properties and drug-likeness of the designed derivatives and confirm the respective stabilities, molecular dynamics (MD) simulations and MM/GBSA (Molecular Mechanics-Generalized Born Surface Area) were used. Table 3 provides the results obtained from the covalent docking of the selected molecules.

**TABLE 3:** The *in silico* designed compounds with their affinity (kcal/mol).

No	Affinity (Kcal/mol)	No	Affinity (Kcal/mol)
14	−5.86	35	−5.45
15	−5.18	36	−4.86
16	−3.74	37	−4.93
17	−5.2	38	−4.94
18	−5.51	39	−4.8
19	−5.42	40	−4.39
20	−4.59	41	−8.17
21	−8.85	42	−6.38
22	−6.15	43	−10.17
23	−5.22	44	−8.64
24	−5.34	45	−8,99
28	−4.83	46	−10.28
29	−4.87	47	−9.17
31	−5.22	48	−9.53
32	−4	49	−9.03
33	−4.25	50	−7.48
34	−4.65	Tofacitinib	−8.57

### Covalent docking analysis of top complexes with high affinity

**FIGURE 8:**
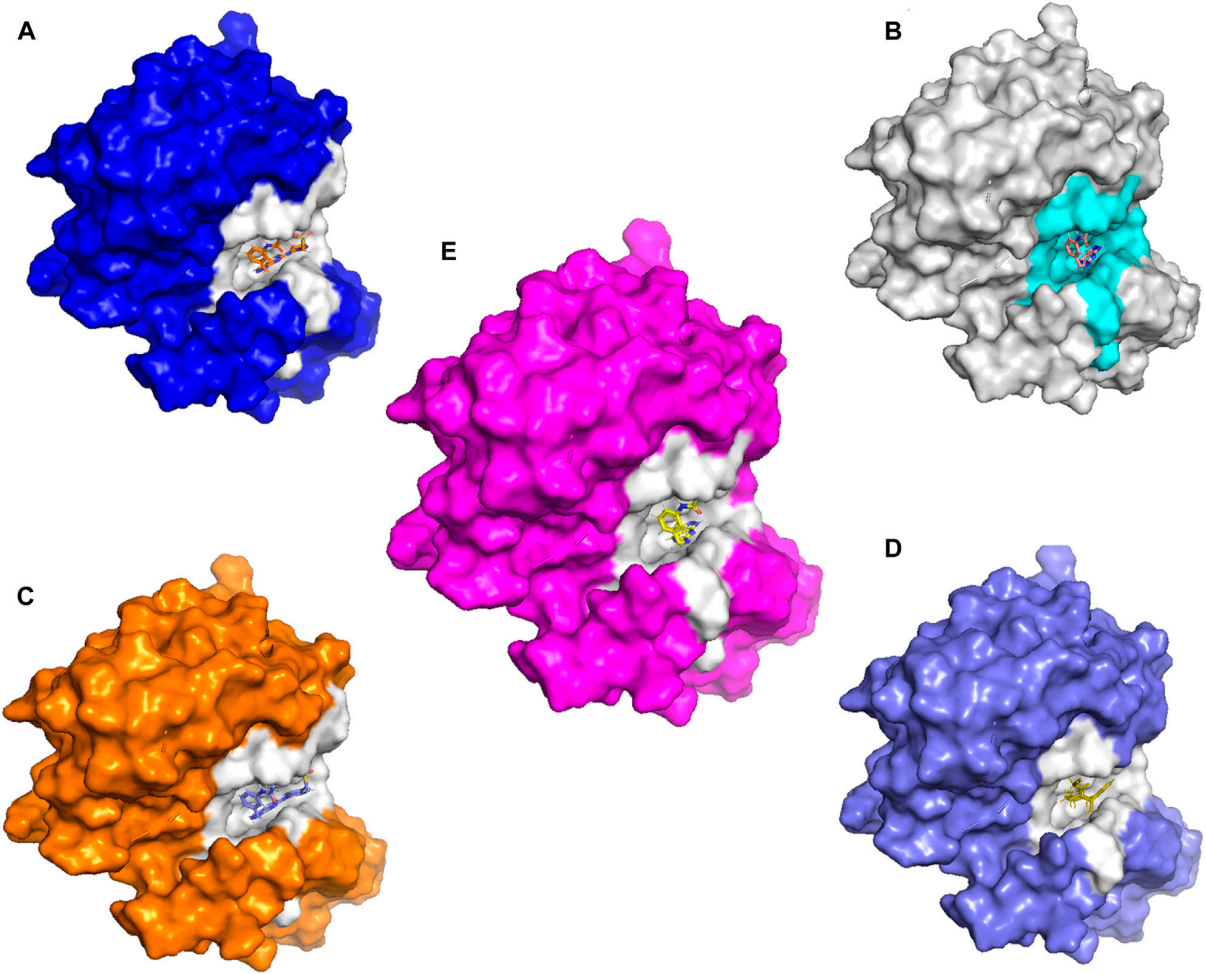
The new compounds in the active site. **(A)**: L21. **(B)**: L41. **(C)**:43. **(D)**:46. **(E)**: Tofacitinib drug.

Molecular docking and covalent docking are two different methods for predicting the interaction between a target molecule and a ligand. Tofacitinib is a reversible inhibitor of JAK kinases. This implies that it does not require the formation of a covalent bond to inhibit its target’s activity. Covalent docking is typically used for molecules that have groups that favour the formation of irreversible bonds with their target. In this study, the target is the ligand-Cys909 (residue). The new compounds designed for covalent docking contain groups that are favourable for this method. Figure 8 shows the different modes and conformations of the ligand with the target during simulation, highlighting the best hot spots for binding to the target.

**FIGURE 9:**
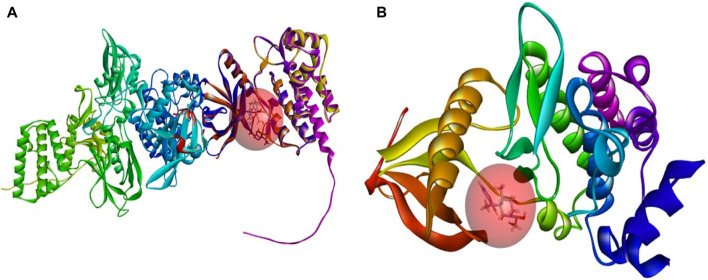
**(A)** The newly designed ligands on the active site of the JAK3 protein modeled. **(B)** Tofacitinib on the active site of the JAK3 reference protein (ID: 4Z16). Tofacitinib on the active site of the JAK3 reference protein (ID: 4Z16).

Covalent docking, more precisely covalent docking with acryldehyde, produces a Michael addition reaction between the Cys909 residue of JAK3 and a ligand containing acryldehyde. This method permits a covalent link to form between the ligand and the protein, resulting in a stable complex. Researchers may use this approach to investigate the binding affinity and specificity of ligands towards the target protein, JAK3, providing useful information for drug development and design in the context of JAK3 inhibition. The covalent docking with acryldehyde through a Michael addition reaction at position Cys909 offers a viable path for the creation of new JAK3-targeting medicinal drugs (Figures 7–9).

The analysis of covalent docking for new inhibitors allowed us to understand their reactivity in the active site corresponding to their affinity. The new compounds are known for the presence of non-covalent bonds of different types, with the presence of a key covalent bond with residue Cys909, considered the therapeutic target for the discovery of new drugs for rheumatoid arthritis and autoimmune diseases. Compound 21 is known for having three hydrogen bonds, two with Leu905 and one with Leu828 and ARG953, and 10 pi-alkyl bonds, one with Leu905 and ALA966, two with ALA853 and LEU828, and three with LEU956, as well as a carbon-hydrogen bond with GLU903. Compound 41 is known for having three hydrogen bonds, one with Leu905, Leu828, and ARG953, and 11 alkyl and pi-alkyl bonds, one with ARG911, Leu905, and ARG953, Val836, two with Ala853 and Leu828, three with Leu956, and a carbon-hydrogen bond with Tyr904 and Glu903.

**FIGURE 10:**
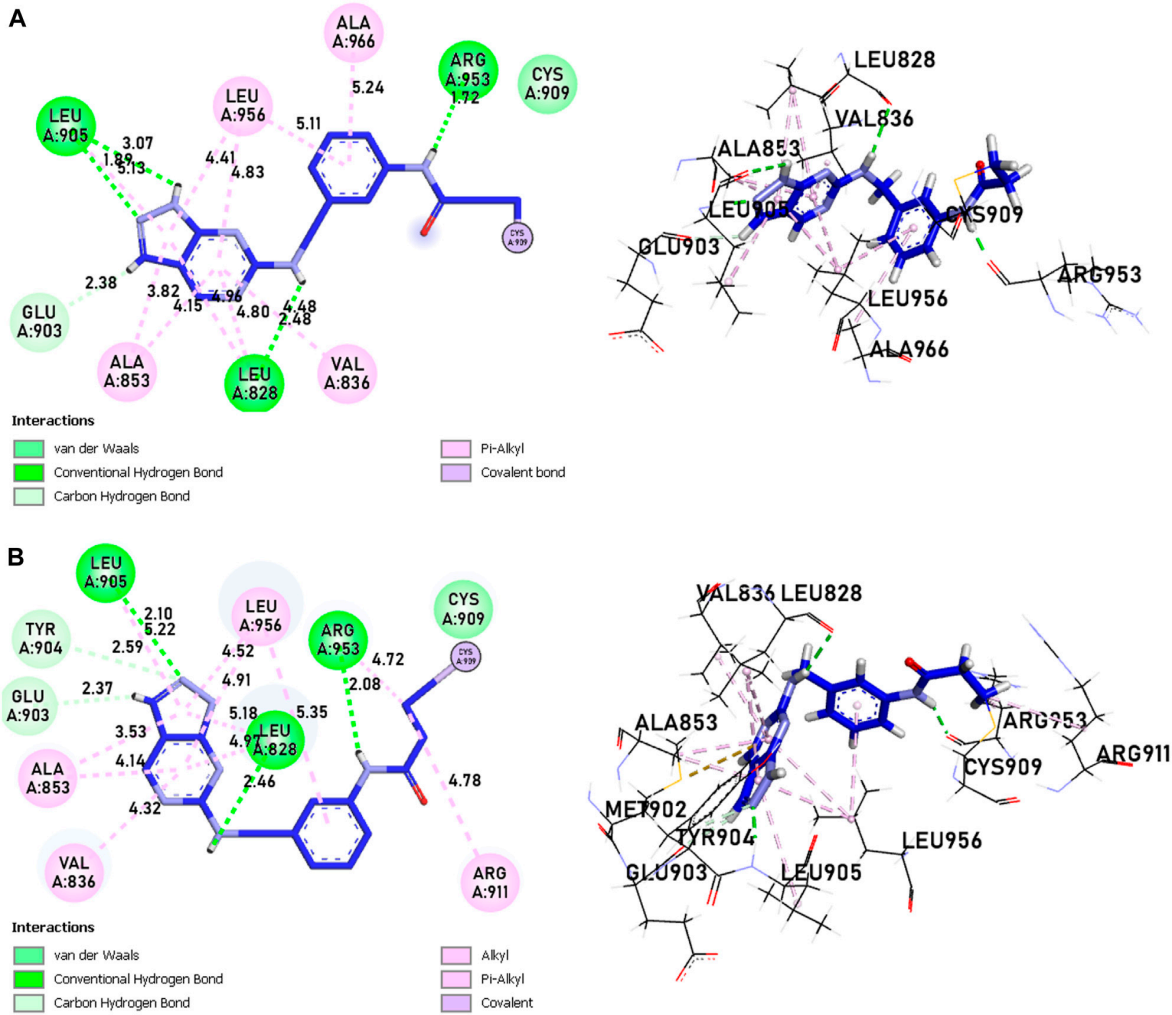
The interactions between the ligands [**(A)** 21 and **(B)** 41] and the JAK3 protein involve 2D and 3D non-covalent bonds, while the interaction between the ligand and Cys909 forms a covalent bond.

**FIGURE 11:**
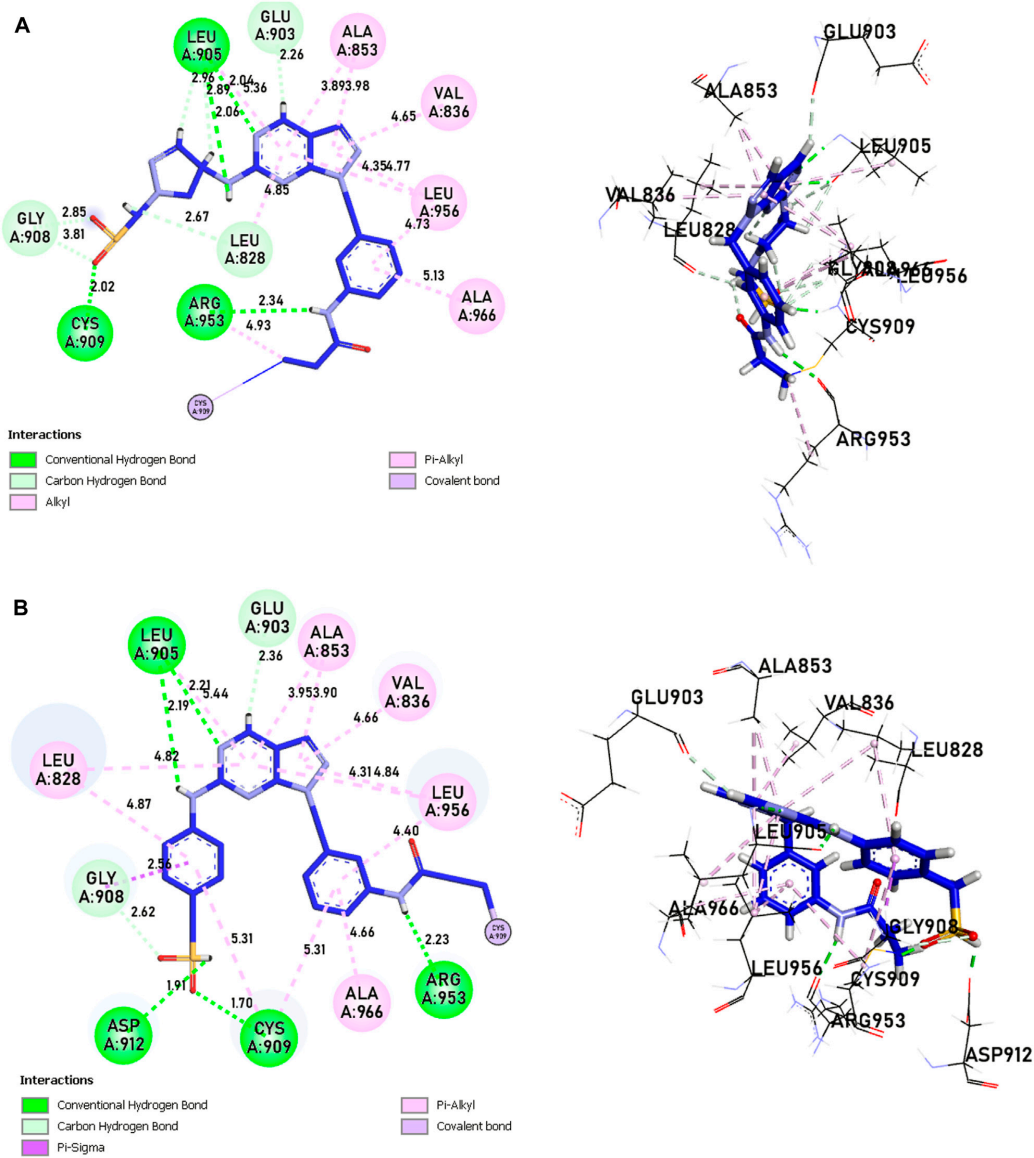
The interactions between the ligands [**(A)** L43 and **(B)** L46]-JAK3 protein involve 2D and 3D non-covalent bonds, while the interaction between the ligand and Cys909 forms a covalent bond.

Compounds 21 and 41 (Figure 10), following development, provide us with powerful new compounds with high affinity by adding SO2 groups, which show remarkable hydrogen bonds as indicated in the molecular docking analysis. Compound 43 (Figure 11) is known for having four hydrogen bonds, two with Leu905, one with Arg953, Leu905, and Cys909, and eight alkyl and pi-alkyl bonds, one with Arg953, Ala966, Val836, two with Ala853, three with Leu956, and a carbon-hydrogen bond with Gly908. Compound 46 (Figure 11) is known for having five hydrogen bonds, one with Arg953, Asp912, and Cys909, and two with Leu905, and 12 pi-alkyl bonds, one with Ala966, Cys909, Leu905, Val836, two with Leu828, Ala853, Cys909, three with Leu956, a carbon-hydrogen bond with Gly908, and a pi-sigma bond with Gly908.

**FIGURE 12:**
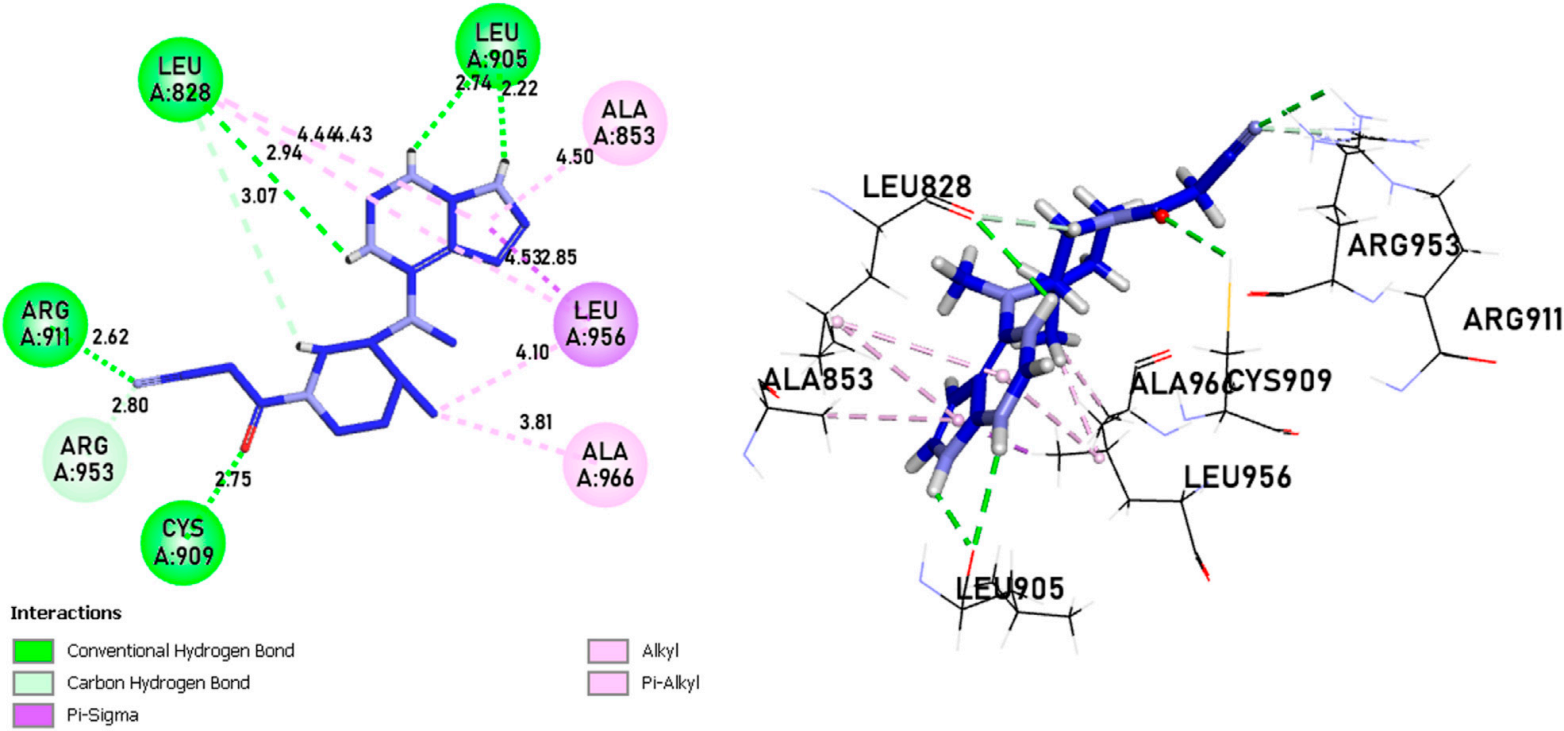
The interactions between Tofacitinib and JAK3 protein involve 2D and 3D non-covalent bonds, while the interaction between the ligand and Cys909 forms a covalent bond.

In comparison to the approved tofacitinib (Figure 12), observed interactions include five hydrogen bonds, one with Leu828, Arg911, Cys909, two with Leu905, alkyl and pi-alkyl bonds, one with Ala966, Ala853, two with Leu956, Leu828, and a pi-sigma bond with Leu956. The presence of hydrogen bonds plays a crucial role in the reactivity of molecules with proteins, influencing the affinity of binding and the potent biological activity. The newly designed molecules exhibit potent inhibition in the presence of hydrogen bonding, extending to the covalent realm.

The analysis of covalent docking for the new inhibitors has allowed us to understand their reactivity at the active site corresponding to their affinity. Compounds 21 (−8.85 kcal/mol) and 41 (−8.17 kcal/mol) already show good affinity for the JAK3 target due to their ability to form multiple hydrogen bonds and non-covalent interactions. The development of these compounds by adding SO2 groups has led to molecules 43 (−10.17 kcal/mol) and 46 (−10.28 kcal/mol) with even higher affinity.

These compounds form more hydrogen bonds, crucial for reactivity with the protein. Although the approved Tofacitinib also exhibits favourable interactions, the new compounds 43 and 46 appear to be more potent inhibitors based on their lower binding energy.

### Drug likeness properties of selected compounds

The newly designed molecules adhere to Lipinski’s rule, but only compounds **21**, **41, 43,** and **46** were subjected to further study, as they have not exhibited Ames’s toxicity. Considering these preliminary results, compounds **21**, **41, 43,** and **46**, together with Tofacitinib as a reference compound, were further investigated (Table 4) using ADMELab 2.0 platform. The FDA-approved drug Tofacitinib is recommended for the treatment of inflammatory disorders such as rheumatoid arthritis, psoriatic arthritis, and ulcerative colitis. The newly designed molecules, which follow Lipinski’s guidelines, work by suppressing specific immunological signalling pathways, leading to a successful reduction of inflammation and alleviation of associated symptoms.

**TABLE 4:** Drug likeness properties of compounds **21**, **41, 43, 46** and Tofacitinib.

Compounds	LogD	Lipinski rule^a^				Lipinski violations^a^	TPSA	nRot
		MW(g/mol)	Log P	nHBA	nHBD			
21	2.487	372.030	2.132	7	2	Accepted	84.730	6
41	2.170	294.120	1.869	7	3	Accepted	95.590	6
43	0.276	442.15	0.246	11	2	Accepted	137.7	8
46	2.46	450.15	2.237	9	2	Accepted	122.1	8
TOFACITINIB	1.716	312.170	1.226	7	1	Accepted	88.910	4

^a^ Prediction ADMElab, 2.0 platforms. LogD, LogP at physiological pH7.4, Optimal 1-3; MW, molecular weight, Optimal 100-600; LogP, Log of octanol/water partition coefficient, Optimal 0-3; nHBA, number of hydrogen bond acceptors, Optimal 0-12; nHBD, number of hydrogen bond donors, Optimal 0-7; Lipinski Violations, MW ≤ 500, logP ≤5, nHBA ≤10, nHBD ≤5. If two properties are out of range, poor absorption or permeability is possible, one is acceptable; TPSA , topological polar surface area, Optimal 0-140; nRot, Number of rotatable bonds, Optimal 0-11.

As reported in Ta, all selected compounds do not violate Lipinski’s rule, suggesting that they could be easily absorbed after oral administration since the Lipinski criteria were respected. The drug-likeness rule proposed by Veber evaluates the number of rotatable bonds (Rb) and the topological polar surface area (TSPA) to predict the ability of drugs to cross the cellular membrane from the gastrointestinal tract. Following Veber’s rule, the number of Rot should be less than 10, and TSPA should be TSPA < 140 and Rb < 10. All molecules reported in Ta respect these parameters, suggesting that they are endowed with good permeable properties.

### ADMET analysis

The evaluation of drug permeability using Caco-2 cell lines serves as a crucial substitute for *in vivo* conditions. Their similarity to the human intestinal epithelium is indispensable for selecting promising drug candidates in development. Caco-2, with a value > −5.15 log cm/s, suggests proper permeability. A Caco-2 value implies that all molecules are present, and those with absorption greater than 30% suggest good absorption, indicating overall favourable molecule absorption. Distribution parameters suggest that all compounds are poorly distributed to the brain (Log BBB < −1), but compound 21 and tofacitinib can penetrate the CNS having Log PS > −2. The highest VDss value (volume of distribution at steady state in humans. Volume Distribution (VD) is a crucial metric describing *in vivo* drug distribution by connecting the administered dose to the initial concentration. VD values aid in predicting properties like plasma protein binding, body fluid distribution, and tissue uptake. A VD in the range of 0.04–20 L/kg suggests a correct VD for most compounds, except for compound 21, which exhibits a lower VD of 0.01. In terms of metabolism, as CYP3A4 is a crucial enzyme, the treatment of autoimmune diseases such as RA and cancer consequently involves the analysis of ADMET. Compounds L21 and L41 only inhibit CYP3A2, while compound L43 acts as a substrate and inhibits CYP3A4. On the other hand, compound L46 is a substrate for CYP3A4 and an inhibitor of CY21C19, CY21C9, and CYP3A4. In contrast, Tofacitinib inhibits CYP3A2. None of the substances have kidney OCT-2 substrate activity, and none have demonstrated AMES mutagenic activity. Regarding toxicity, none of the compounds have been linked to skin sensitivity, but all compounds, including tofacitinib, have been associated with hepatotoxicity, except for compound 21, which does not exhibit this toxicity.

Taking into account all the gathered data, the newly designed compounds (Table 5) display favorable drug-like properties, presenting a robust pharmacokinetic profile that ensures efficient oral absorption and suitable distribution in tissues.

**TABLE 5:** pkCSM predictions for compounds 21, 41, and Tofacitinib.

ADMET			41	21	43	46	Tofacitinib
Absorption	Water solubility	(Log mol/L)	−2.999	−2.892	−2.953	−4.029	−3.526
	Caco-2 permeability	(Log P_app_, 10^−6^ cm/s)	0.534	1.23	0.633	0.796	1.36
	Intestinal absorption (human)		69.134	89.671	72.856	**79.729**	93.481
Distribution	VDss (human)	(Log L/kg)	1.141	0.011	0.268	0.013	0.402
	Fraction unbound (human)	(Fu)	0.339	0.381	0.058	0	0.41
	BBB permeability	(Log BBB)	−1.482	−0.029	−1.291	**−1.282**	−0.752
	CNS permeability	(Log PS)	−4.556	−1.429	−3.311	−3.549	−0.752
Metabolism	CY21D6	Substrate	No	No	No	No	No
	CYP3A4		No	No	Yes	Yes	No
	CY41A2	Inhibitor	Yes	Yes	No	No	Yes
	CY21C19		No	No	No	Yes	No
	CY21C9		No	No	No	Yes	No
	CY21D6		No	No	No	No	No
	CYP3A4		No	No	Yes	Yes	No
Excretion	Total Clearance	(Log mL/min/kg)	0.105	−16.253	0.466	0.262	0.848
	Renal OCT2 substrate	(Yes/No)	No	No	No	No	No
	AMES		No	No	No	No	No
Toxicity	Skin Sensitization		No	No	No	No	No
	Hepatotoxicity		Yes	No	Yes	Yes	Yes

### Molecular dynamics (MD) simulation analysis

**FIGURE 13:**
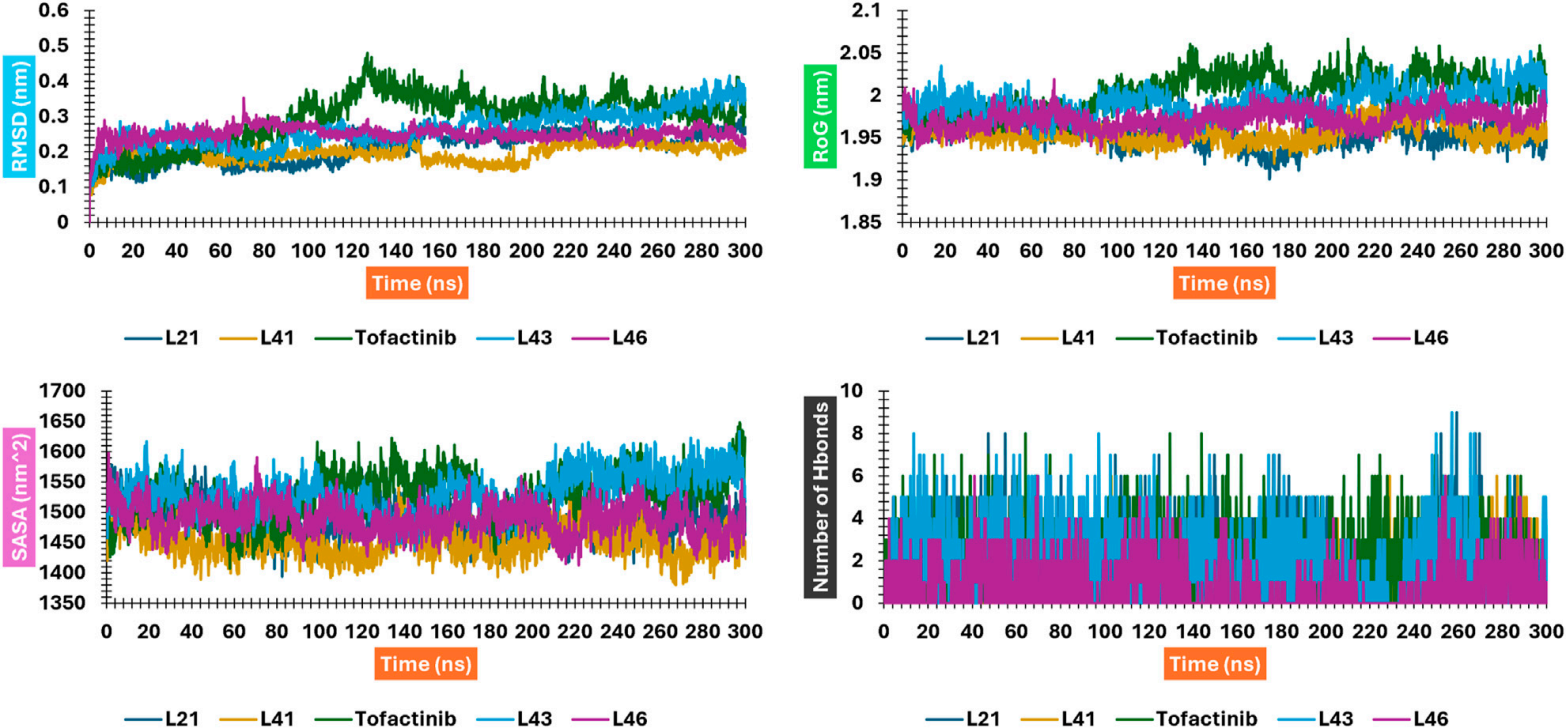
RMSD, RMSF, SASA, and RoG plots for compounds **41** and **21** interacting with JAK3; Figure is not mentioned in the text.

**FIGURE 14:**
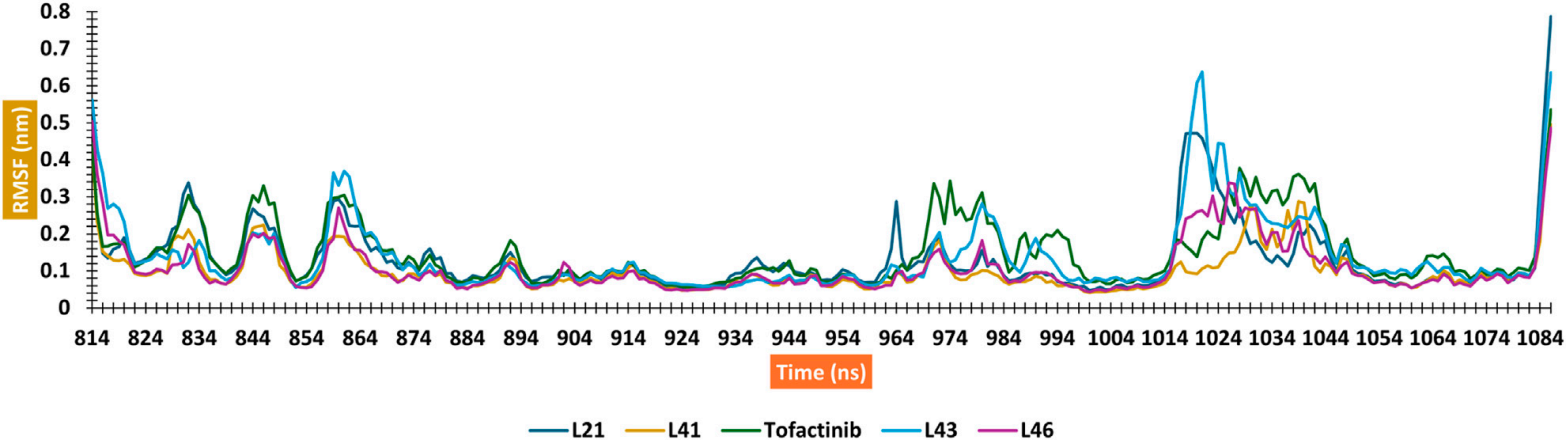
Shows H-bond plots for compounds 21 and 41 interacting with JAK3.

**FIGURE 15:**
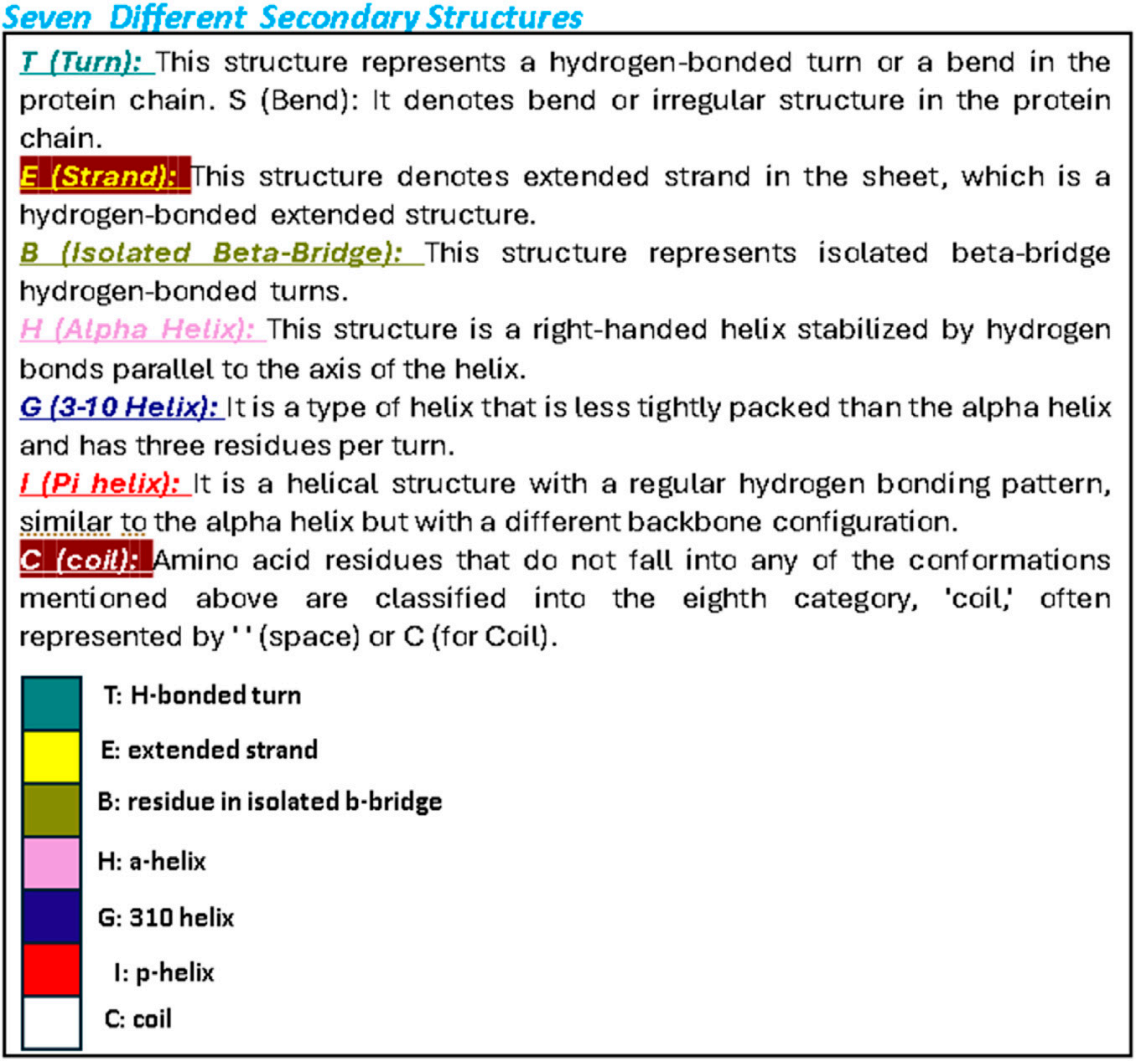
Letter codes.

**FIGURE 16:**
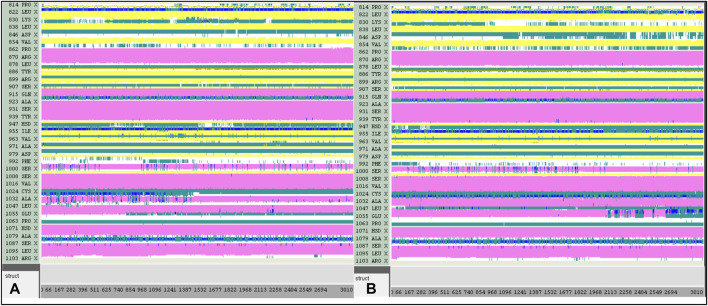
DSSP analysis was performed during the 300 ns simulation. **(A)**: 21-JAK3. **(B)**: 41-JAK3.

**FIGURE 17:**
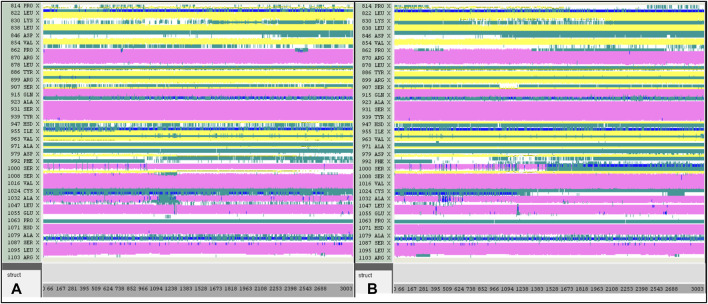
DSSP analysis was performed during the 300 ns simulation. **(A)**: 43-JAK3. **(B)**: 46-JAK3.

**FIGURE 18:**
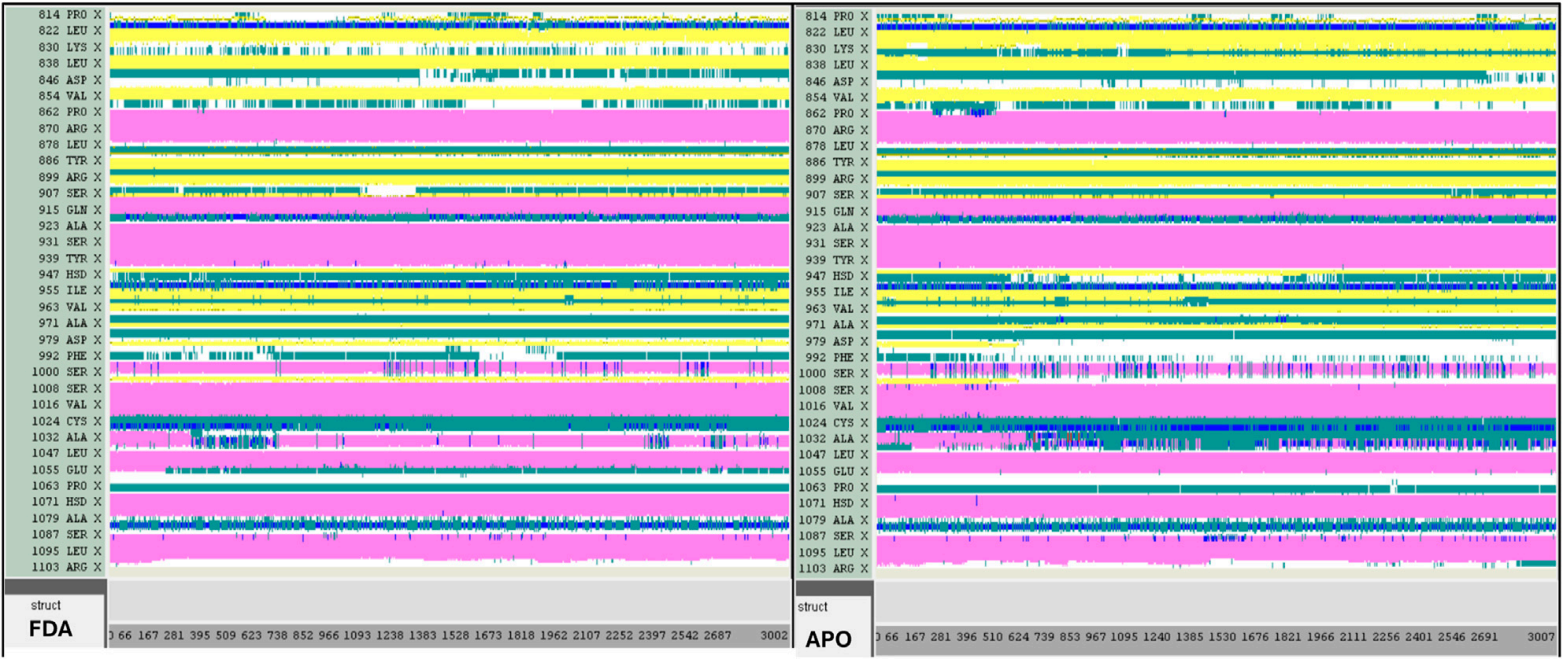
DSSP analysis was performed during the 300 ns simulation for the Tofacitinib-JAK3 and Apo-protein.

**FIGURE 19:**
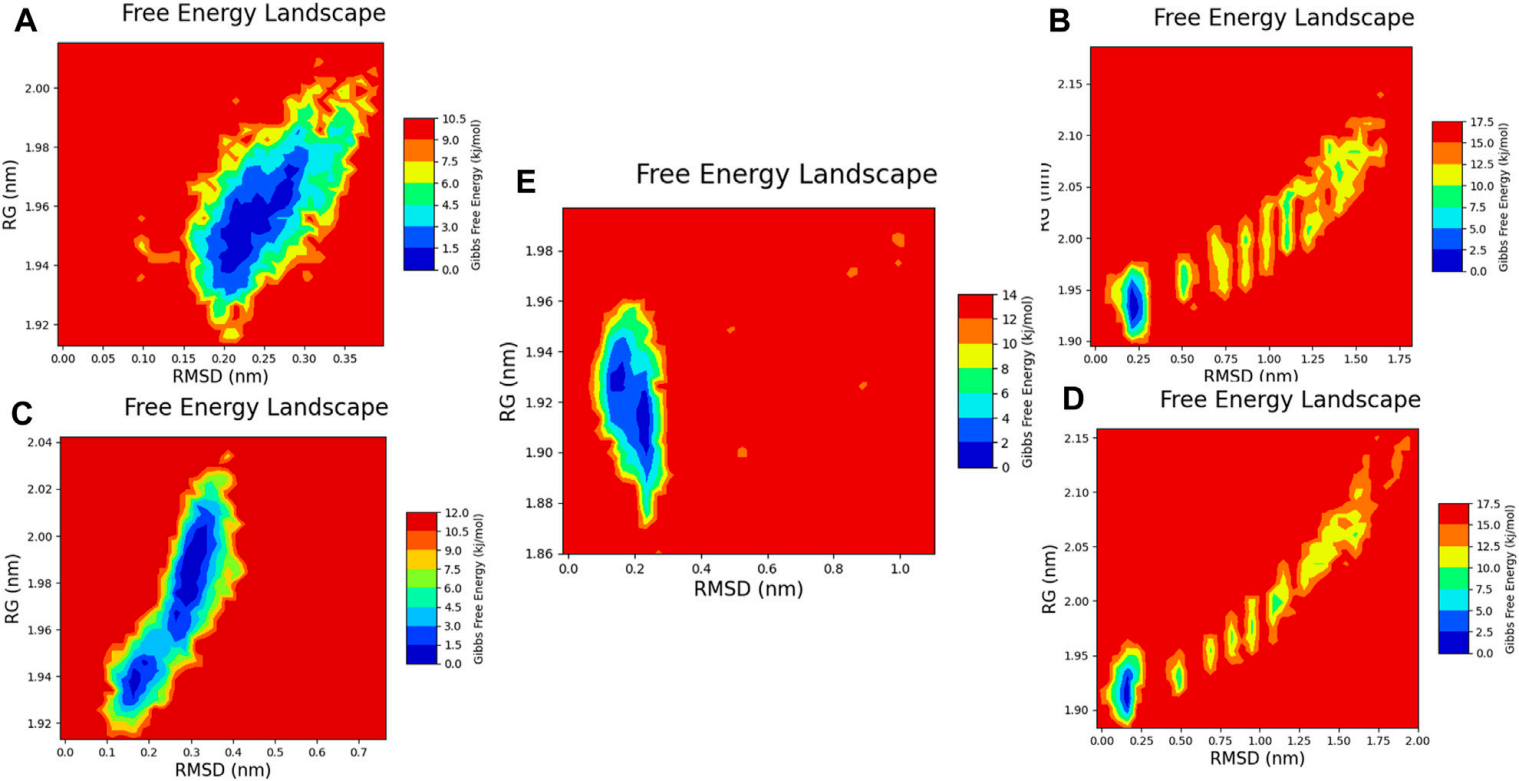
Free energy 2D landscape (FEL) diagram. **(A)** Composed of 21. **(B)** Composed of 41. **(C)** Composed of 43. **(D)** Composed of 46. **(E)** Tofacitinib.

**FIGURE 20:**
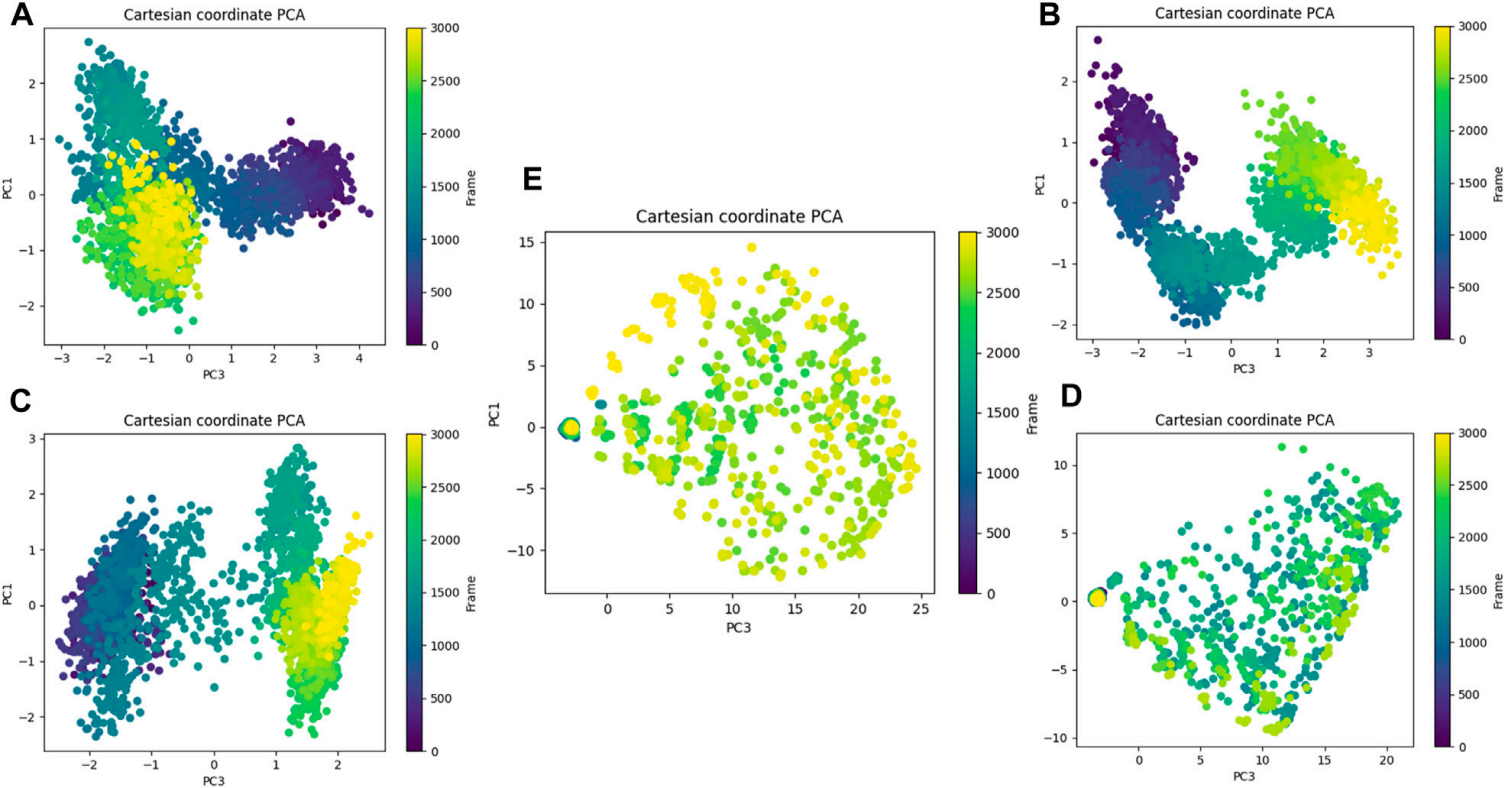
Principal component analysis. **(A)** Composed of 21. **(B)** Composed of 41. **(C)** Composed of 43. **(D)** Composed of 46. **(E)** Tofacitinib.

**FIGURE 21:**
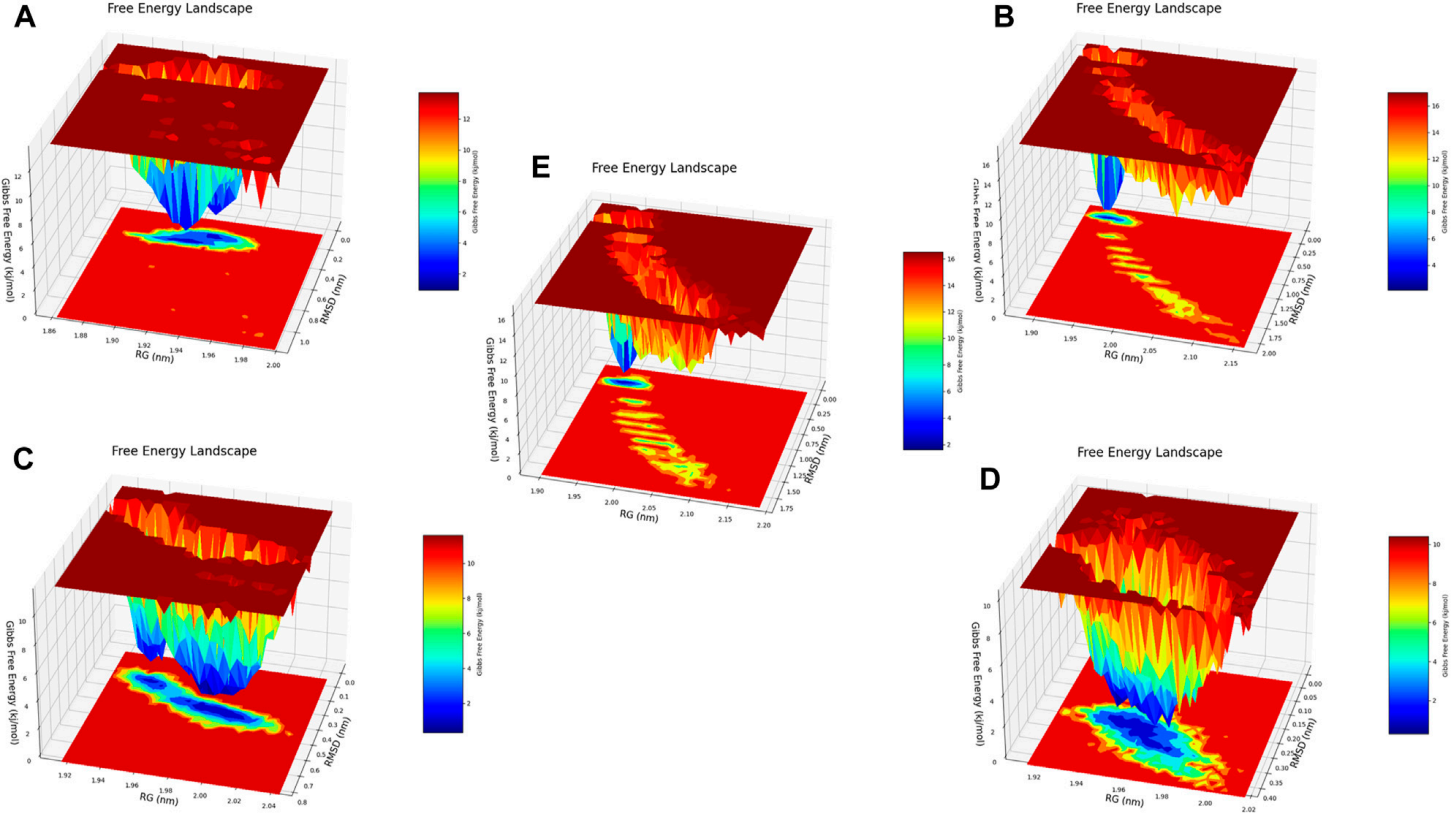
Free energy 3D landscape (FEL) diagram. **(A)** Composed of 21. **(B)** Composed of 41. **(C)** Composed of 43. **(D)** Composed of 46. **(E)** Tofacitinib.

**FIGURE 22:**
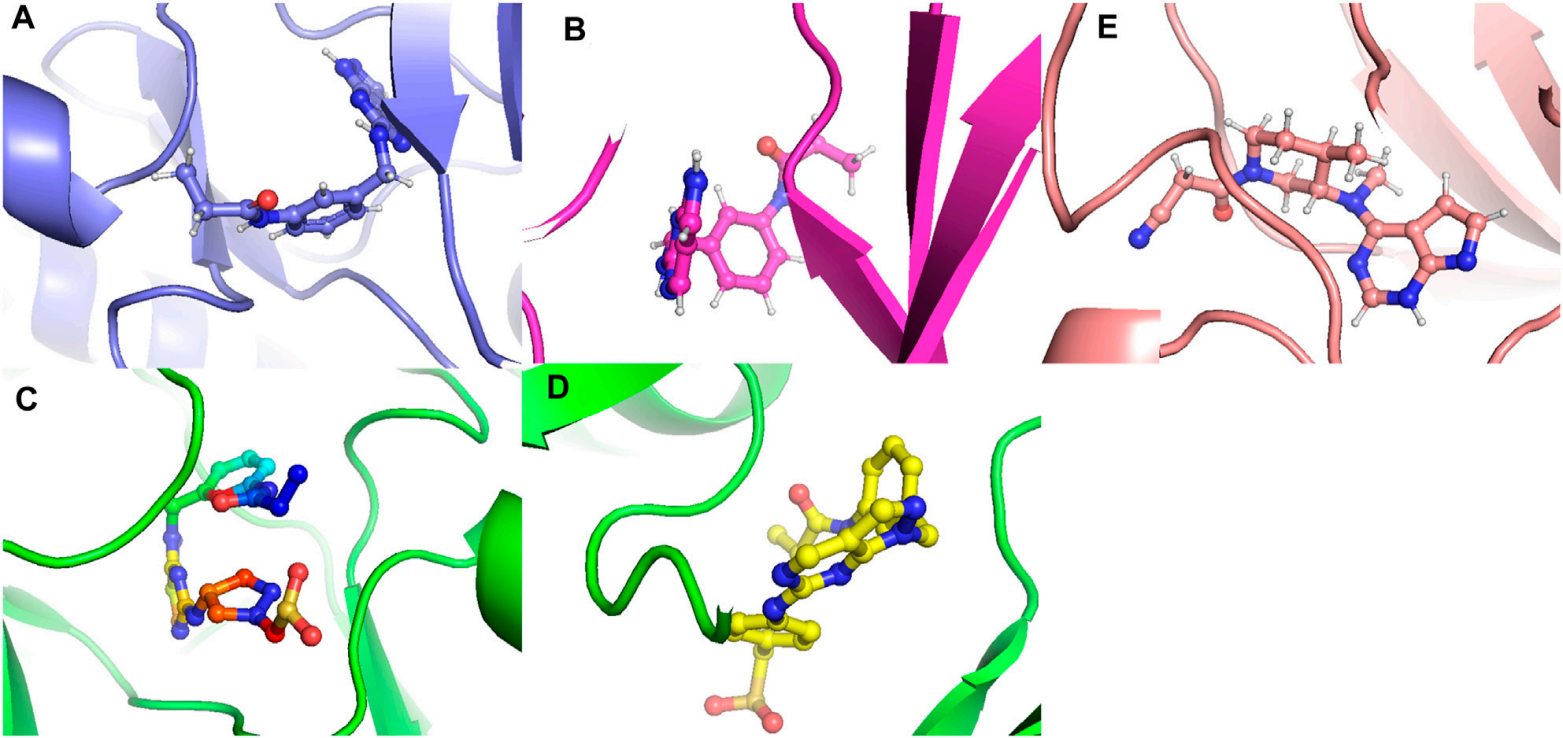
The conformations that favor minimum energy. **(A)** Composed of 21. **(B)** Composed of 41. **(C)** Composed of 43. **(D)** Composed of 46. **(E)** Tofacitinib.

A computer technique for simulating the actual physical motions of atoms and molecules is called MD simulation. It offers details on the alterations in protein structure that occur throughout time. Protein dynamics, folding, stability, and interactions may all be studied using MD. MD simulations give a dynamic representation of how proteins behave throughout time. One may gain a thorough knowledge of protein stability, flexibility, compactness, solvent exposure, and secondary structural changes by analysing RMSD, RMSF, RoG, SASA, H-bonds, DSSP, Fel, and PCA from MD trajectories (Figures 13–22).

The RMSD analysis suggests a variation between the initial and final positions of the ligand during a 300 ns simulation. The results in Figure 13 show that compounds L21, L41, and L46 A experienced an RMSD ranging between 0.1 nm and 0.2. For compound L43 and Tofacitinib, the RMSD ranged from 0.1 to 0.4. The analysis of the RMSD results suggests stability in the following order: L46, L21, L41, L43, and Tofacitinib. The results of RoG have values ranging between 1.95 and 2 (compounds L46, L21, L41, L43) nm, and for Tofacitinib, they are between 1.95 and 2.05 nm. For SASA, the values are between 1,400 and 1,550 (compounds L46, L21, L41, L43) nm^2^ and for Tofacitinib between 1,450 and 1,600 nm^2^. The results of RoG and SASA suggest a compactness of the complexes for the newly designed molecules during the simulation, which is better than the Tofacitinib drug. The analysis of H-bonds shows that the compounds exhibit hydrogen interactions ranging from a minimum of one to a maximum of eight during an H-bond simulation, suggesting their high-affinity stability. The analysis of RMSF presents suitable results for the flexibility of residues for each complex. The RMSF results suggest stable residue movements for compounds L21 and L46 compared to the other complexes, confirming favourable stability for Ligands (L21 and L41) in interaction with JAK3 during a 300 ns simulation.

Contrasted with the apo-protein JAK3, the DSSP analysis (Figures 15–18) of complexes involving Ligands (L21, L41, L43, L46, and Tofacitinib) and JAK3 (Figures 13, 14) revealed that the recently designed compounds exhibited robust structural integrity during the simulation. In comparison to the apo-protein JAK3, the most prominent transitions observed in all three complexes were Coil and Turn, Turn, Extended strand, Coil with 3–10 helix, turn, and Alpha helix. These findings suggest the potential of the newly developed compounds as JAK3 inhibitors, as they consistently maintained stable secondary structures throughout the simulation.

### Principal component analysis and FEL analysis

The free energy landscapes are very useful for interpreting and analysing biomolecular processes such as molecular folding, aggregation, and recognition.

The analysis conducted by Fel and PCA (Figures 19–21) suggests that the conformations occurring during the 300 ns energy minima are as follows: For compound 21, a Radius of Gyration (RoG) of 1.90 nm and Root Mean Square Deviation (RMSD) of 0.20 nm were observed, with a PCA distribution of components (PC1 and PC1) ranging between (−2) -(+2) and (−3) -(+4). For compound 41, a RoG of 1.95 nm and RMSD of 0.25 Å were observed, along with a PCA distribution of components (PC1 and PC1) situated between (−2) -(+2) and (−3) -(+3). For compound 43, two minima were identified. The first exhibited a RoG of 1.94 nm and RMSD of 0.15 Å, while the second displayed a RoG of 1.98 nm and RMSD of 0.3 nm. The PCA distribution of components (PC1 and PC1) for the second minimum is situated between (−2) -(+3) and (−2) -(+2). For compound 46, a RoG of 1.91 nm and RMSD of 0.22 Å were observed, along with a PCA distribution of components (PC1 and PC1) ranging between (−10) -(+10) and (0) -(+20). Finally, for Tofacitinib, two minima were identified. The first exhibited a RoG of 1.91 nm and RMSD of 0.20 Å, while the second displayed a RoG of 1.93 nm and RMSD of 0.19 nm. The PCA distribution of components (PC1 and PC1) for the second minimum is situated between (−15) -(+15) and (0) -(+25). The analysis of Fel and PCA allows for defining the confirmations that occur and the minimum energy present in Figure 22.

### MM/GBSA analysis

The MM/GBSA analysis is an important step in the design of new target protein inhibitors, particularly in the field of drug discovery. This technique enables the energetic stability of protein-ligand complexes to be assessed and the binding strength between the two molecules to be predicted. It thus complements molecular docking and molecular dynamics analyses by providing a more accurate estimate of the affinity and stability of the complexes formed. This approach can help researchers select the most promising molecules for further study and guide the design of new compounds with improved affinity and energetic stability.

**FIGURE 23:**
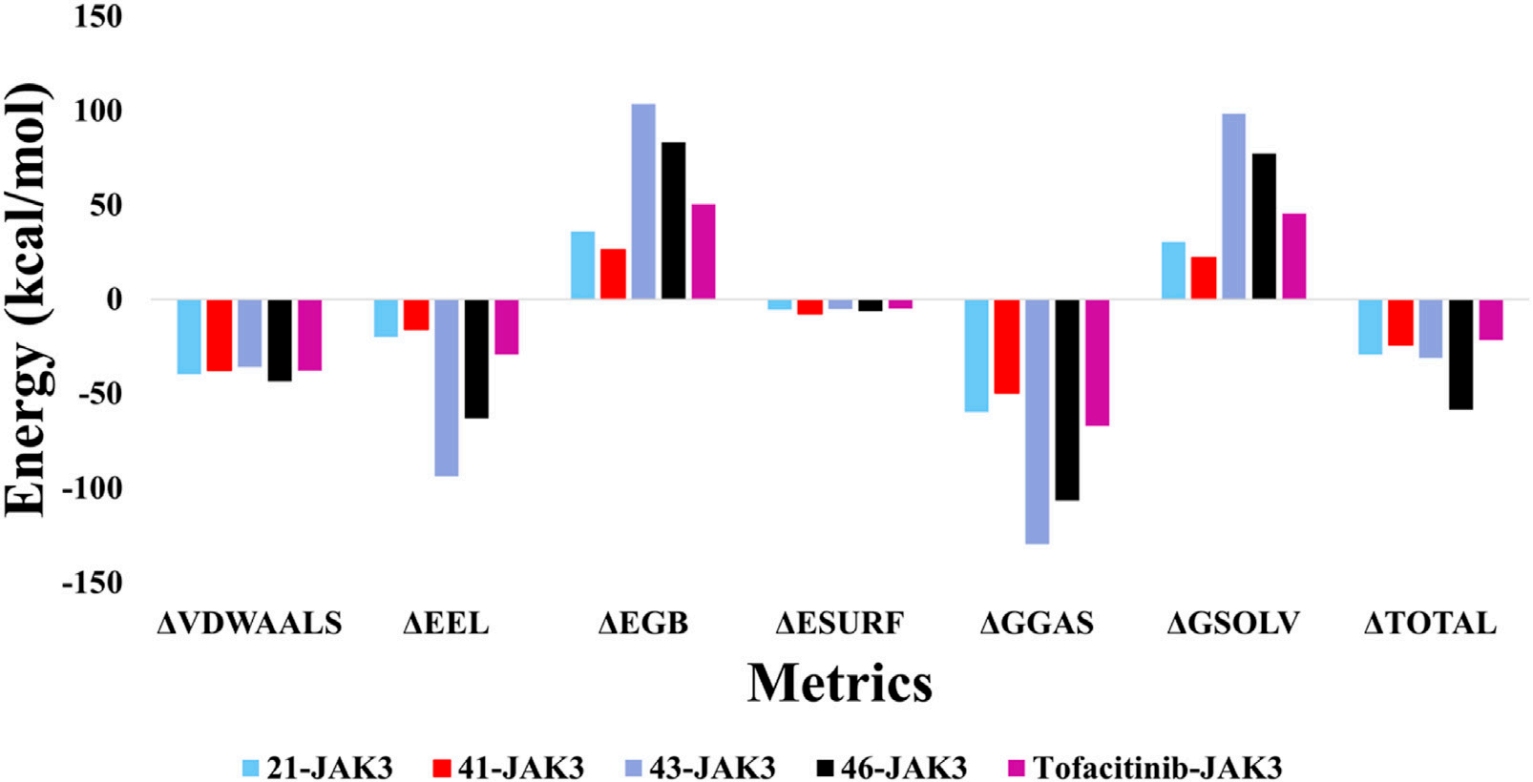
The results of calculating free binding energy using MM/GBSA.

The MM/GBSA findings for the complexes involving ligand-JAK3 reveal significant total binding free energy (Δ_TOTAL_) values of −24.14 and −58.29 kcal/mol, respectively, in comparison to the tofacitinib-JAK3 complex (Figure 23). Various energy factors were considered when determining these energies. The ΔVDWAALS term signifies van der Waals energy, which is consistently negative across all ligand-protein complexes, suggesting an attractive interaction between the ligand and JAK3 protein atoms. The ΔEEL term, representing electrostatic energy, is also negative, indicating a favourable interaction between the positive and negative charges of the ligand and protein. The ΔEGB term, corresponding to Born solvation energy, is positive, indicating system desolvation upon complex formation. The ΔESURF term, denoting surface energy, is negative, implying a reduction in the surface exposure of molecules upon complex formation. The ΔGGAS term, reflecting the gas-phase energy of the protein-ligand complex, is negative, pointing to a robust ligand-JAK3 affinity. Lastly, the ΔGSOLV term, representing the solvation energy of the protein-ligand complex, is positive, indicating favourable solvation of the systems.

Collectively, these outcomes indicate that newly introduced ligands, in conjunction with the JAK3 protein, exhibit a robust affinity and enhanced energy stability when compared to the tofacitinib-JAK3 complex. This observation suggests their potential as effective JAK3 inhibitors. These findings hold promise and underscore the potential of these recently formulated compounds as inhibitors of JAK3.

## Conclusion

To discover new pyrazolopyrimidine derivatives as JAK3 inhibitors based on the most active molecule with a pIC50 = 10 discovered in a previous study, this research used homology modelling, virtual screening, ADMET analysis, covalent docking, molecular dynamics (MD) simulation, and MM/GBSA. Previously, the three-dimensional structure of the JAK3 protein was developed using homology modelling. Then, the MM2 force field was used to extract and reduce the compound set initially. Using AutoDock 4.2, molecules from a set of 41 were selected for further screening. The compounds were initially evaluated by ADMET analysis based on the molecules having no AMES toxicity. The molecular docking results based on affinity and the ADMET results based on no AMES toxicity suggested molecules L21 and L41 for further study. Furthermore, to develop the latest compound based on the addition of SO_2,_ which presents favourable interactions, substituents were added to different electrostatic, steric, hydrophobic, hydrogen bond donor, and acceptor groups to obtain good compounds among the new nine designs by taking the two best with high affinity (kcal/mol), allowing the selection of good molecules L43 and L46. The stability evaluation of the best-ranked compounds L21 and 41 in the first phase and L43 and L46 in the second phase in the JAK3 protein receptor binding pocket was conducted using MD simulation and MMGBSA binding free energy calculations considering that tofacitinib, an FDA-approved drug for the treatment of rheumatoid arthritis, as the reference from the initial docking instant to the instant t. Consequently, this research led to the discovery of new potent inhibitors. In conclusion, using various computational approaches, our work was effective in identifying new pyrazolopyrimidine derivatives as potential JAK3 inhibitors. However, further studies are needed to confirm the effectiveness of these compounds as therapeutic agents, including *in vitro* and *in vivo* testing. The computational strategy used in this work can be an efficient tool in the rational development of new drugs for various disorders.

## Data Availability

The datasets presented in this study can be found in online repositories. The names of the repository/repositories and accession number(s) can be found in the article/Supplementary Material.
