# Supplementary material for: Computational insights into rational design and virtual screening of pyrazolopyrimidine derivatives targeting Janus kinase 3 (JAK3)
Source: Front Chem. 2024 Aug 12;12:1425220. doi: 10.3389/fchem.2024.1425220 (PMC11345245; doi:10.3389/fchem.2024.1425220)
Supplement: Supplementary file 1 [file DataSheet1.pdf]

Table S1 Comparative Analysis of Molecular Dynamics Metrics.

RMSD	$\mathrm{RMSD}(t) = \left[\frac{1}{M}\sum_{i=1}^{N} m_i \left|\mathbf{r}_i(t) - \mathbf{r}_i^{\mathrm{ref}}\right|^2\right]^{\frac{1}{2}}$, Equation 1	$M = \Sigma_i m_i$ and $\mathbf{r}_i(t)$ is the position of atom *i* at time *t* after least square fitting the structure to the reference structure.
RMSF	$\mathrm{RMSF_i} = \left[\frac{1}{\mathrm{T}}\sum_{\mathrm{t_j}=1}^{\mathrm{T}} \left|\mathbf{r}_\mathrm{i}(\mathrm{t_j}) - \mathbf{r}_\mathrm{i}^{\mathrm{ref}}\right|^2\right]^{1/2}$, Equation 2	*T* is the time over which one wants to average, and **ref** is the reference position of particle *i*. This reference position will be the time-averaged position of the same particle *i*.
Fel and PCA	$\Delta G = -k_b \ln \mathrm{P}(CV1, CV2)$	$\boldsymbol{k_B}$ and ***T*** are the Boltzmann constant and absolute temperature, respectively, and ***P* (*CV*$_1$, *CV*$_2$)** is the probability distribution of the molecular system along the reaction coordinates/ collective variables (*CV*$_1$ and *CV*$_2$).

Table S2 Energy Decomposition Analysis in MM/GBSA Binding.

Van der Waals energy variation	$\Delta_{\mathrm{VDWAALS}} = \sum^i\sum^j 6\varepsilon\left[\left(\frac{\sigma_{ij}}{r_{ij}}\right)^{12} - 2\left(\frac{\sigma_{ij}}{r_{ij}}\right)^{6}\right]$, Equation 3.	ε is the energy scaling factor, σ_{ij} is the distance at which the potential energy of the interaction between atoms i and j is zero, rij is the distance between atoms i and j, and the summations are over all pairs of atoms i and j. The equation is based on the Lennard-Jones potential.
Electrostatic energy variation	$\Delta \mathrm{E_{EL}} = \sum^i\sum^j \frac{q_i * q_j}{\varepsilon \mathrm{r}}$, Equation 4.	q_i and q_j are the partial charges on atoms i and j, r is the distance between atoms i and j, ε is the dielectric constant of the solvent, and the summations are over all pairs of atoms i and j. The equation is based on Coulomb's law.
Generalized Born energy variation	$\Delta \mathrm{E_{GB}} = \gamma\sum^i \frac{q_i^2}{r_i} + k\sum^i\sum^j \frac{q_i * q_j}{r_{ij}} + \sum^i \sigma_i$, Equation 5.	γ and κ are constants that depend on the solvent dielectric constant and ionic strength, qi is the partial charge on atom i, r_i is the distance from atom i to the center of the solvent-accessible surface, r_{ij} is the distance between atoms i and j, and σ_i is a surface tension term that penalizes the creation of a solvent-accessible surface. The equation is based on the Generalized Born model.
Surface energy variation	$\Delta \mathrm{E_{SURF}} = \gamma\sum^i \frac{1}{r_i}$, Equation 6.	γ is a constant that depends on the solvent dielectric constant and ionic strength, and r_i is the distance from atom i to the centre of the solvent-accessible surface. The equation is based on the solvent-accessible surface area (SASA) model.
Energy variation in the gas phase	$\Delta \mathrm{G_{GAS}} = \mathrm{H} - \mathrm{TS}$, Equation 7.	H is the enthalpy, T is the temperature, and S is the entropy. The equation is derived from the Gibbs-Helmholtz equation.
Solvation-free energy variation	ΔG_{SOLV} = ΔHSOLV - TΔSSOLV (6) $\Delta \mathrm{G_{SOLV}} = \Delta \mathrm{H_{SOLV}} - \mathrm{T}\Delta \mathrm{S_{SOLV}}$, Equation 8.	ΔH_{SOLV} is the enthalpy change associated with the solvation process, ΔS_{SOLV} is the entropy change associated with the solvation process, and T is the temperature. The equation is based on the thermodynamic definition of Gibbs free energy.
